# Supplementary material for: Longitudinal investigation of spatial memory and retinal parameters in a 5xFAD model of Alzheimer’s disease reveals differences dependent on genotype and sex
Source: Biomed Opt Express. 2025 Dec 19;17(1):405–26. doi: 10.1364/BOE.579020 (PMC12795427; doi:10.1364/BOE.579020)
Supplement: Supplementary file 1 [file boe-17-1-405-s001.pdf]

Supplemental Document

Biomedical Optics EXPRESS

Longitudinal investigation of spatial memory and retinal parameters in a 5xFAD model of Alzheimer's disease reveals differences dependent on genotype and sex: supplement

GEORG LADURNER,[1,2,*] CONRAD W. MERKLE,[1] LUCAS MAY,[1] SYBREN WORM,[1] YASH PATEL,[1] MARIA VARAKA,[1] MAGDALENA DAURER,[2] LAURENZ JAUK,[2] ROLAND RABL,[2] PHILIPP KÖNIGSHOFER,[3] GERHARD GARHÖFER,[4] MANUELA PROKESCH,[2] AND BERNHARD BAUMANN[1,5]

[1]*Medical University of Vienna, Center for Medical Physics and Biomedical Engineering, Währinger Gürtel 18-20, 1090 Vienna, Austria*
[2]*Scantox Neuro GmbH, Parkring 12, 8074 Grambach, Austria*
[3]*Medical University of Vienna, Core Facility Laboratory Animal Breeding and Husbandry, Währinger Gürtel 18-20, 1090 Vienna, Austria*
[4]*Medical University of Vienna, Department of Clinical Pharmacology, Währinger Gürtel 18-20, 1090 Vienna, Austria*
[5]*Medical University of Innsbruck, Institute of Biomedical Physics, Müllerstraße 44, 6020 Innsbruck, Austria*
[*]*georg.ladurner@meduniwien.ac.at*

This supplement published with Optica Publishing Group on 19 December 2025 by The Authors under the terms of the Creative Commons Attribution 4.0 License in the format provided by the authors and unedited. Further distribution of this work must maintain attribution to the author(s) and the published article's title, journal citation, and DOI.

Supplement DOI: https://doi.org/10.6084/m9.figshare.30580391

Parent Article DOI: https://doi.org/10.1364/BOE.579020

Longitudinal investigation of spatial memory and retinal parameters in a 5xFAD model of Alzheimer's disease reveals differences dependent on genotype and sex: Supplemental document

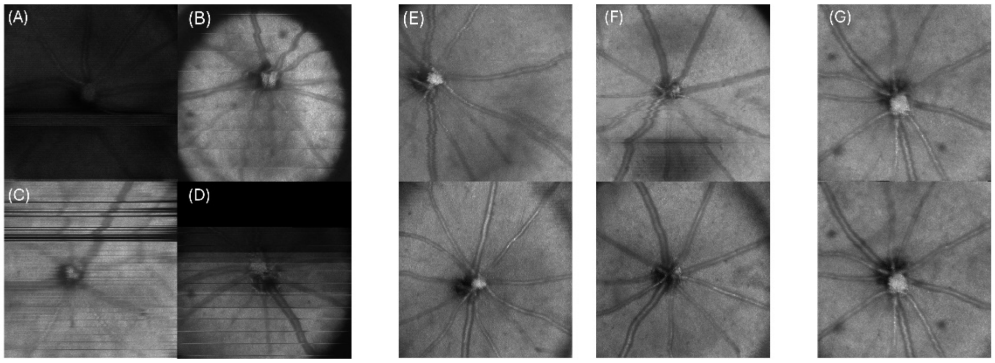

Supplementary Figure 1. (A-D) Scans excluded because of insufficient quality. (A) Low signal, (B) strong vignetting, (C) strong eye motion, and (D) only partially visible field of view. (E-G) Examples of decision making based on pairs of volume scans from three eyes. (E) Top scan is strongly off center, so here the bottom scan was chosen. (F) The top scan is distorted by motion artifacts, so here the bottom scan was chosen. (G) Both scans are of comparable quality, thus in this case, the resulting parameters were averaged.

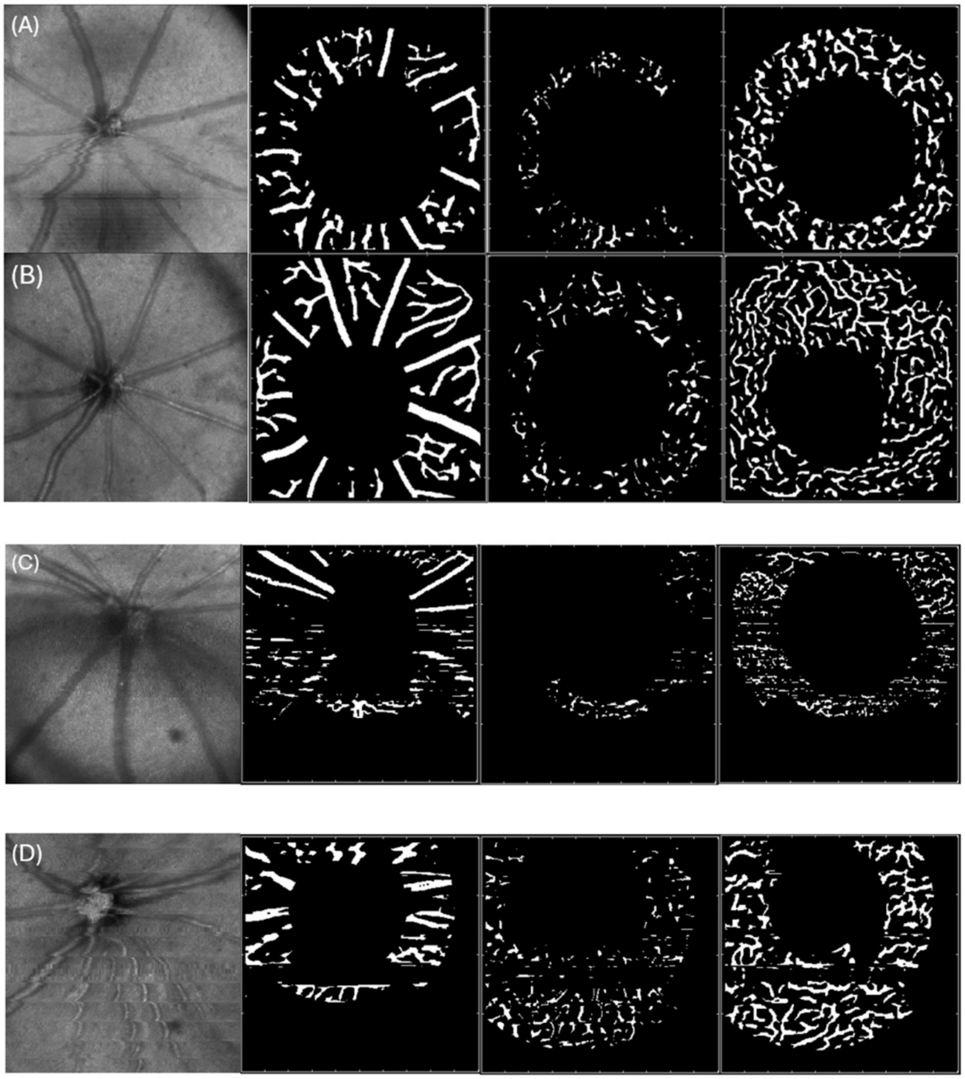

Supplementary Figure 2. Image artifacts and corresponding OCTA data. (A-B) Two examples from the same eye (from left to right): en-face reflectivity images of the same eye and the corresponding OCTA segmentation of SVP, ICP and DCP. For this example, the volume scan displayed in panel (B) was chosen for OCTA data analysis due to less motion and better SNR. (C-D) Exemplary scans excluded from the data analysis: The data shown in panel (C) was excluded due to bad signal quality, while those in panel (D) were excluded due to strong motion and thus unreliable segmentation.

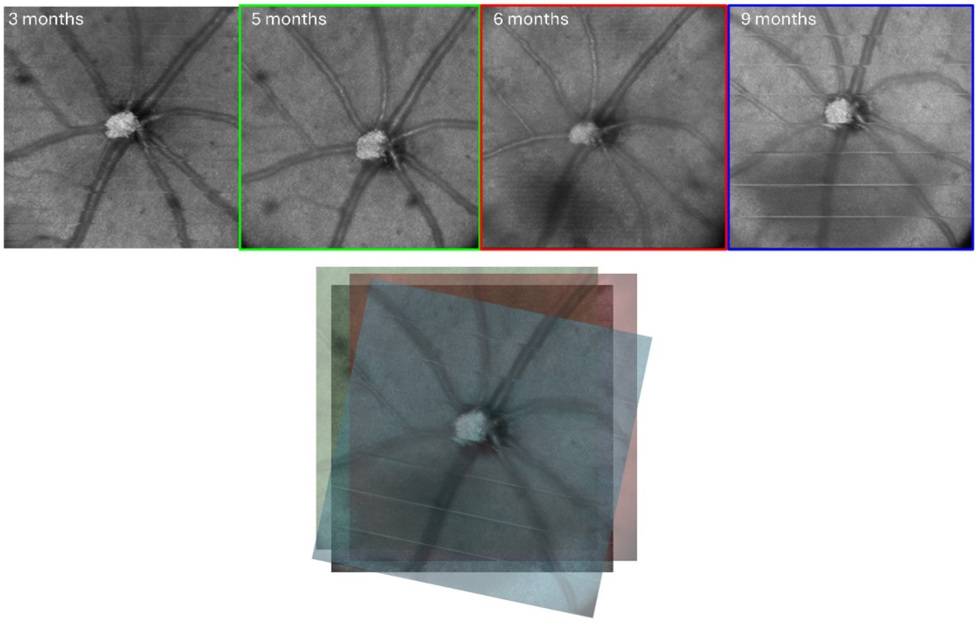

Supplementary Figure 3. Visualization of overlapping OCT fundus data for four different timepoints. The top row shows the individual fundus projection images while beneath, a visualization of their overlap after rigid image registration and stacking is provided.

Supplementary Table 1. Total number of used scans, excluded scans and percentage of excluded scans for each measurement.

Age [weeks]	12	20	24	36	Total
**Total number of acquired scans**	135	91	96	59	381
**Number of used scans**	125	87	86	50	348
**Number of excluded scans due to quality**	7	3	9	4	23
**Number of duplicate scans excluded**	3	1	1	5	10
**Total number of excluded scans**	10	4	10	9	33
**Percentage of excluded scans**	7.4 %	4.4%	10.4 %	15.3 %	8.7 %
**Percentage of excluded scans without duplicate**	5.2 %	3.3 %	9.4 %	6.8 %	5.3 %

Supplementary Table 2. Number of individual volumetric OCTA datasets used for angiography, excluded from the analysis and percentage of excluded scans

Age [weeks]		12		20		24		36		Total	
**Number of acquired volume scans**		135		91		96		59		381	
**Number of analyzed volume scans**		121		84		85		50		340	
**Transgenic**	**Non-transgenic**	64	57	41	43	43	42	25	25	173	165
**Number of excluded scans due to quality**		11		6		10		4		33	
**Number of duplicate scans excluded**		3		1		1		5		10	
**Total number of excluded scans**		16		7		11		9		43	
**Percentage of excluded scans**		11.8 %		7.7 %		11.5 %		15.3 %		11.2 %	
**Percentage of excluded scans without duplicate**		8.1%		6.6 %		10.4 %		6.8 %		8.7 %	

Composite Learning Index

A learning index was calculated for each parameter as

$Z_i = \frac{X_i - Mean(X_i)}{Sted(X_i)}$. (1)

where Z_i = learning score for variable i and X_i= measured value for parameter i (e.g., latency, floating). Next, the composite learning index was calculated as the sum of the learning indices normalized by the number of parameters:

$Z = \frac{1}{k}\sum_{i=1}^{k} Z_i$. (2)

The learning indices for each parameter (floating, latency, distance swam, velocity and thigmotaxis) were calculated for each testing day, while time spent in target quadrant and number of target zone crossings, were included into the index for day 5. Thereby, negative results for the composite learning index for individual mice indicate above average parameters. For ntg males, an index of 0.059 was calculated, whereas for tg male, the index was -0.020. For ntg and tg females, the composite learning index was calculated as -0.106 and 0.013, respectively. No significant differences between the groups were observed.

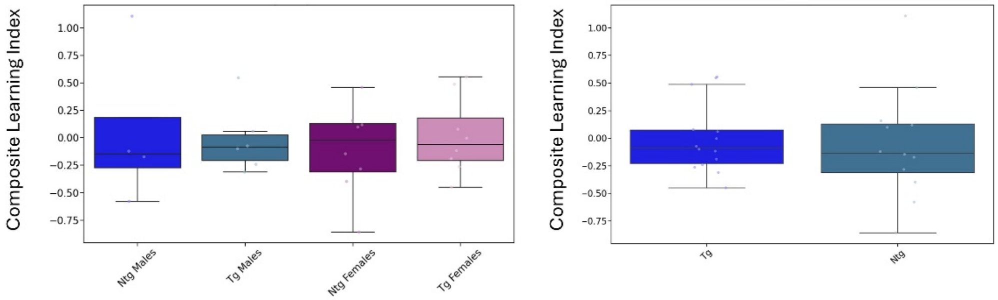

Supplementary Figure 4. (A) Composite learning index for ntg and tg male, and ntg and tg female mice (B). Composite leaning index for tg and ntg mice.

Correlation Coefficient	Weight	SVP Density	ICP Density	DCP Density	Total Retina	IRL	RPE	RNFL	IPL	INL	ORL	PRC	OPL	Number of Target Zone Crossings	% of time in sector with platform	Latency on Day 3	Latency on Day 4	Distance swam (m)	Velocity (m/s)	Thigmotaxis %	Floating %
Weight		0.4691	0.4559	0.1468	0.5539	0.6622	0.3746	0.2949	0.2008	0.2965	0.3379	0.2580	0.4342	0.0662	0.1680	-0.1660	-0.3125	-0.0326	0.2966	0.1352	0.1759
SVP Density	0.4691		-0.3331	0.3683	-0.1106	0.0498	-0.2922	-0.1968	0.1824	0.3125	-0.2897	-0.2467	-0.3246	-0.0086	0.0656	0.2185	-0.1113	-0.0652	0.0637	0.0873	-0.0749
ICP Density	0.4559	-0.3331		-0.0330	0.6091	0.5695	0.5694	0.6591	-0.2693	0.1428	0.5562	0.4435	0.7159	0.4801	0.3075	-0.5473	-0.1948	0.0514	0.3050	0.2867	0.3654
DCP Density	0.1468	0.3683	-0.0330		-0.0457	0.0846	-0.0688	-0.4394	0.5011	0.2060	-0.1925	-0.1855	-0.1299	0.3408	0.2487	0.6155	0.2802	0.0953	-0.0547	0.0882	-0.2940
Total Retina	0.5539	-0.1106	0.6091	-0.0457		0.9390	0.5162	0.4975	0.1211	0.6429	0.9136	0.8664	0.6593	0.4771	0.5064	-0.1828	-0.3014	-0.0879	0.0716	0.0237	0.1558
IRL	0.6622	0.0498	0.5695	0.0846	0.9390		0.4275	0.3654	0.2993	0.7235	0.7180	0.6635	0.5713	0.5359	0.6156	-0.1142	-0.2953	-0.0919	0.0985	0.0360	0.1632
RPE	0.3746	-0.2922	0.5694	-0.0688	0.5162	0.4275		0.6623	-0.2680	-0.1900	0.5392	0.3279	0.9484	-0.0065	0.0598	-0.3602	-0.3865	-0.2407	0.0782	-0.2154	0.2483
RNFL	0.2949	-0.1968	0.6591	-0.4394	0.4975	0.3654	0.6623		-0.7577	-0.0306	0.5749	0.4524	0.6979	0.1095	0.1424	-0.5604	-0.2934	-0.0766	0.1457	0.0294	0.3725
IPL	0.2008	0.1824	-0.2693	0.5011	0.1211	0.2993	-0.2680	-0.7577		0.3847	-0.1088	-0.0576	-0.2176	0.1775	0.1858	0.4417	0.0551	-0.0123	-0.0563	-0.0457	-0.2389
INL	0.2965	0.3125	0.1428	0.2060	0.6429	0.7235	-0.1900	-0.0306	0.3847		0.4456	0.5452	-0.0520	0.6221	0.7133	0.2217	-0.0257	0.0310	-0.0371	0.1395	-0.0632
ORL	0.3379	-0.2897	0.5562	-0.1925	0.9136	0.7180	0.5392	0.5749	-0.1088	0.4456		0.9690	0.6589	0.3318	0.2970	-0.2349	-0.2609	-0.0692	0.0285	0.0054	0.1223
PRC	0.2580	-0.2467	0.4435	-0.1855	0.8664	0.6635	0.3279	0.4524	-0.0576	0.5452	0.9690		0.4528	0.3606	0.3130	-0.1637	-0.1614	0.0049	0.0116	0.0693	0.0338
OPL	0.4342	-0.3246	0.7159	-0.1299	0.6593	0.5713	0.9484	0.6979	-0.2176	-0.0520	0.6589	0.4528		0.0995	0.1191	-0.3496	-0.4507	-0.2649	0.0674	-0.1917	0.3389
Number of Target Zone Crossing	0.0662	-0.0086	0.4801	0.3408	0.4771	0.5359	-0.0065	0.1095	0.1775	0.6221	0.3318	0.3606	0.0995		0.8193	0.0878	0.2531	0.2756	0.2147	0.4503	-0.1840
% of time in sector with platform	0.1680	0.0656	0.3075	0.2487	0.5064	0.6156	0.0598	0.1424	0.1858	0.7133	0.2970	0.3130	0.1191	0.8193		0.0316	0.0699	0.1192	0.1138	0.3242	-0.1524
Latency on Day 3	-0.1660	0.2185	-0.5473	0.6155	-0.1828	-0.1142	-0.3602	-0.5604	0.4417	0.2217	-0.2349	-0.1637	-0.3496	0.0878	0.0316		0.4163	0.1043	-0.2631	-0.1299	-0.4141
Latency on Day 4	-0.3125	-0.1113	-0.1948	0.2802	-0.3014	-0.2953	-0.3865	-0.2934	0.0551	-0.0257	-0.2609	-0.1614	-0.4507	0.2531	0.0699	0.4163		0.8990	0.5430	0.6638	-0.8409
Distance swam (m)	-0.0326	-0.0652	0.0514	0.0953	-0.0879	-0.0919	-0.2407	-0.0766	-0.0123	0.0310	-0.0692	0.0049	-0.2649	0.2756	0.1192	0.1043	0.8990		0.8338	0.8775	-0.8141
Velocity (m/s)	0.2966	0.0637	0.3050	-0.0547	0.0716	0.0985	0.0782	0.1457	-0.0563	-0.0371	0.0285	0.0116	0.0674	0.2147	0.1138	-0.2631	0.5430	0.8338		0.8738	-0.5950
Thigmotaxis %	0.1352	0.0873	0.2867	0.0882	0.0237	0.0360	-0.2154	0.0294	-0.0457	0.1395	0.0054	0.0693	-0.1917	0.4503	0.3242	-0.1299	0.6638	0.8775	0.8738		-0.6370
Floating %	0.1759	-0.0749	0.3654	-0.2940	0.1558	0.1632	0.2483	0.3725	-0.2389	-0.0632	0.1223	0.0338	0.3389	-0.1840	-0.1524	-0.4141	-0.8409	-0.8141	-0.5950	-0.6370	

P-Value	Weight	SVP Density	ICP Density	DCP Density	Total Retina	IRL	RPE	RNFL	IPL	INL	ORL	PRC	OPL	Number of Target Zone Crossings	% of time in sector with platform	Latency on Day 3	Latency on Day 4	Distance swam (m)	Velocity (m/s)	Thigmotaxis %	Floating %
Weight		0.1058	0.1364	0.6166	0.0399	0.0099	0.1869	0.3061	0.4912	0.3033	0.2374	0.3731	0.1209	0.8221	0.5658	0.5706	0.2766	0.9118	0.3031	0.6450	0.5475
SVP Density	0.1058		0.2900	0.2156	0.7192	0.8717	0.3327	0.5193	0.5510	0.2985	0.3370	0.4165	0.2791	0.9779	0.8313	0.4733	0.7174	0.8324	0.8361	0.7766	0.8080
ICP Density	0.1364	0.2900		0.9189	0.0355	0.0532	0.0533	0.0197	0.3972	0.6580	0.0604	0.1487	0.0088	0.1142	0.3310	0.0655	0.5441	0.8739	0.3351	0.3662	0.2428
DCP Density	0.6166	0.2156	0.9189		0.8768	0.7737	0.8152	0.1159	0.0680	0.4799	0.5097	0.5254	0.6579	0.2330	0.3912	0.0191	0.3320	0.7458	0.8527	0.7643	0.3077
Total Retina	0.0399	0.7192	0.0355	0.8768		0.0000	0.0588	0.0703	0.6800	0.0131	0.0000	0.0001	0.0103	0.0846	0.0646	0.5317	0.2949	0.7652	0.8078	0.9360	0.5947
IRL	0.0099	0.8717	0.0532	0.7737	0.0000		0.1273	0.1990	0.2985	0.0034	0.0038	0.0097	0.0328	0.0482	0.0191	0.6975	0.3054	0.7548	0.7377	0.9029	0.5771
RPE	0.1869	0.3327	0.0533	0.8152	0.0588	0.1273		0.0099	0.3542	0.5152	0.0466	0.2525	0.0000	0.9823	0.8391	0.2058	0.1722	0.4070	0.7904	0.4595	0.3920
RNFL	0.3061	0.5193	0.0197	0.1159	0.0703	0.1990	0.0099		0.0017	0.9172	0.0315	0.1044	0.0055	0.7093	0.6273	0.0371	0.3087	0.7945	0.6193	0.9206	0.1896
IPL	0.4912	0.5510	0.3972	0.0680	0.6800	0.2985	0.3542	0.0017		0.1744	0.7111	0.8450	0.4548	0.5438	0.5247	0.1139	0.8516	0.9666	0.8484	0.8768	0.4107
INL	0.3033	0.2985	0.6580	0.4799	0.0131	0.0034	0.5152	0.9172	0.1744		0.1103	0.0438	0.8598	0.0175	0.0042	0.4463	0.9304	0.9162	0.8998	0.6344	0.8301
ORL	0.2374	0.3370	0.0604	0.5097	0.0000	0.0038	0.0466	0.0315	0.7111	0.1103		0.0000	0.0104	0.2465	0.3025	0.4190	0.3676	0.8142	0.9230	0.9854	0.6769
PRC	0.3731	0.4165	0.1487	0.5254	0.0001	0.0097	0.2525	0.1044	0.8450	0.0438	0.0000		0.1040	0.2053	0.2760	0.5761	0.5816	0.9867	0.9685	0.8139	0.9087
OPL	0.1209	0.2791	0.0088	0.6579	0.0103	0.0328	0.0000	0.0055	0.4548	0.8598	0.0104	0.1040		0.7349	0.6852	0.2205	0.1058	0.3601	0.8188	0.5116	0.2360
Number of Target Zone Crossing	0.8221	0.9779	0.1142	0.2330	0.0846	0.0482	0.9823	0.7093	0.5438	0.0175	0.2465	0.2053	0.7349		0.0003	0.7653	0.3826	0.3401	0.4610	0.1062	0.5288
% of time in sector with platform	0.5658	0.8313	0.3310	0.3912	0.0646	0.0191	0.8391	0.6273	0.5247	0.0042	0.3025	0.2760	0.6852	0.0003		0.9145	0.8124	0.6848	0.6984	0.2581	0.6029
Latency on Day 3	0.5706	0.4733	0.0655	0.0191	0.5317	0.6975	0.2058	0.0371	0.1139	0.4463	0.4190	0.5761	0.2205	0.7653	0.9145		0.1387	0.7228	0.3634	0.6581	0.1411
Latency on Day 4	0.2766	0.7174	0.5441	0.3320	0.2949	0.3054	0.1722	0.3087	0.8516	0.9304	0.3676	0.5816	0.1058	0.3826	0.8124	0.1387		0.0000	0.0448	0.0096	0.0002
Distance swam (m)	0.9118	0.8324	0.8739	0.7458	0.7652	0.7548	0.4070	0.7945	0.9666	0.9162	0.8142	0.9867	0.3601	0.3401	0.6848	0.7228	0.0000		0.0002	0.0000	0.0004
Velocity (m/s)	0.3031	0.8361	0.3351	0.8527	0.8078	0.7377	0.7904	0.6193	0.8484	0.8998	0.9230	0.9685	0.8188	0.4610	0.6984	0.3634	0.0448	0.0002		0.0000	0.0248
Thigmotaxis %	0.6450	0.7766	0.3662	0.7643	0.9360	0.9029	0.4595	0.9206	0.8768	0.6344	0.9854	0.8139	0.5116	0.1062	0.2581	0.6581	0.0096	0.0000	0.0000		0.0143
Floating %	0.5475	0.8080	0.2428	0.3077	0.5947	0.5771	0.3920	0.1896	0.4107	0.8301	0.6769	0.9087	0.2360	0.5288	0.6029	0.1411	0.0002	0.0004	0.0248	0.0143	

Table 3: Correlation Coefficient and corresponding p-value for or all tested parameters for all transgenic animals. For the Correlation coefficient red indicates values from 0-0.3, yellow =0.3-0.5 and green 0.5-1. For the p-value red indicates values above 0.15. yellow values from 0.15-0.1 and green values from below 0.05.

R-Squared	Weight	SVP Density	ICP Density	DCP Density	Total Retina	IRL	RPE	RNFL	IPL	INL	ORL	PRC	OPL	Number of Target Zone Crossings	% of time in sector with platform	Latency on Day 3	Latency on Day 4	Distance swam (m)	Velocity (m/s)	Thigmotaxis %	Floating %
Weight		0.2201	0.2078	0.0215	0.3068	0.4386	0.1403	0.0870	0.0403	0.0879	0.1142	0.0666	0.1885	0.0044	0.0282	0.0276	0.0977	0.0011	0.0880	0.0183	0.0309
SVP Density	0.2201		0.1110	0.1357	0.0122	0.0025	0.0854	0.0387	0.0333	0.0977	0.0839	0.0609	0.1054	0.0001	0.0043	0.0477	0.0124	0.0043	0.0041	0.0076	0.0056
ICP Density	0.2078	0.1110		0.0011	0.3710	0.3244	0.3242	0.4344	0.0725	0.0204	0.3093	0.1967	0.5125	0.2305	0.0945	0.2995	0.0379	0.0026	0.0930	0.0822	0.1335
DCP Density	0.0215	0.1357	0.0011		0.0021	0.0072	0.0047	0.1931	0.2511	0.0424	0.0371	0.0344	0.0169	0.1162	0.0619	0.3788	0.0785	0.0091	0.0030	0.0078	0.0864
Total Retina	0.3068	0.0122	0.3710	0.0021		0.8817	0.2665	0.2475	0.0147	0.4133	0.8346	0.7507	0.4347	0.2276	0.2564	0.0334	0.0909	0.0077	0.0051	0.0006	0.0243
IRL	0.4386	0.0025	0.3244	0.0072	0.8817		0.1828	0.1335	0.0896	0.5235	0.5155	0.4402	0.3264	0.2872	0.3789	0.0130	0.0872	0.0084	0.0097	0.0013	0.0267
RPE	0.1403	0.0854	0.3242	0.0047	0.2665	0.1828		0.4387	0.0718	0.0361	0.2907	0.1075	0.8994	0.0000	0.0036	0.1298	0.1494	0.0580	0.0061	0.0464	0.0617
RNFL	0.0870	0.0387	0.4344	0.1931	0.2475	0.1335	0.4387		0.5741	0.0009	0.3305	0.2046	0.4871	0.0120	0.0203	0.3140	0.0861	0.0059	0.0212	0.0009	0.1388
IPL	0.0403	0.0333	0.0725	0.2511	0.0147	0.0896	0.0718	0.5741		0.1480	0.0118	0.0033	0.0474	0.0315	0.0345	0.1951	0.0030	0.0002	0.0032	0.0021	0.0571
INL	0.0879	0.0977	0.0204	0.0424	0.4133	0.5235	0.0361	0.0009	0.1480		0.1985	0.2972	0.0027	0.3871	0.5088	0.0491	0.0007	0.0010	0.0014	0.0195	0.0040
ORL	0.1142	0.0839	0.3093	0.0371	0.8346	0.5155	0.2907	0.3305	0.0118	0.1985		0.9391	0.4341	0.1101	0.0882	0.0552	0.0681	0.0048	0.0008	0.0000	0.0150
PRC	0.0666	0.0609	0.1967	0.0344	0.7507	0.4402	0.1075	0.2046	0.0033	0.2972	0.9391		0.2050	0.1300	0.0979	0.0268	0.0260	0.0000	0.0001	0.0048	0.0011
OPL	0.1885	0.1054	0.5125	0.0169	0.4347	0.3264	0.8994	0.4871	0.0474	0.0027	0.4341	0.2050		0.0099	0.0142	0.1222	0.2031	0.0702	0.0045	0.0367	0.1148
Number of Target Zone Crossing	0.0044	0.0001	0.2305	0.1162	0.2276	0.2872	0.0000	0.0120	0.0315	0.3871	0.1101	0.1300	0.0099		0.6712	0.0077	0.0641	0.0760	0.0461	0.2027	0.0339
% of time in sector with platform	0.0282	0.0043	0.0945	0.0619	0.2564	0.3789	0.0036	0.0203	0.0345	0.5088	0.0882	0.0979	0.0142	0.6712		0.0010	0.0049	0.0142	0.0130	0.1051	0.0232
Latency on Day 3	0.0276	0.0477	0.2995	0.3788	0.0334	0.0130	0.1298	0.3140	0.1951	0.0491	0.0552	0.0268	0.1222	0.0077	0.0010		0.1733	0.0109	0.0692	0.0169	0.1715
Latency on Day 4	0.0977	0.0124	0.0379	0.0785	0.0909	0.0872	0.1494	0.0861	0.0030	0.0007	0.0681	0.0260	0.2031	0.0641	0.0049	0.1733		0.8081	0.2948	0.4407	0.7071
Distance swam (m)	0.0011	0.0043	0.0026	0.0091	0.0077	0.0084	0.0580	0.0059	0.0002	0.0010	0.0048	0.0000	0.0702	0.0760	0.0142	0.0109	0.8081		0.6953	0.7700	0.6627
Velocity (m/s)	0.0880	0.0041	0.0930	0.0030	0.0051	0.0097	0.0061	0.0212	0.0032	0.0014	0.0008	0.0001	0.0045	0.0461	0.0130	0.0692	0.2948	0.6953		0.7636	0.3540
Thigmotaxis %	0.0183	0.0076	0.0822	0.0078	0.0006	0.0013	0.0464	0.0009	0.0021	0.0195	0.0000	0.0048	0.0367	0.2027	0.1051	0.0169	0.4407	0.7700	0.7636		0.4057
Floating %	0.0309	0.0056	0.1335	0.0864	0.0243	0.0267	0.0617	0.1388	0.0571	0.0040	0.0150	0.0011	0.1148	0.0339	0.0232	0.1715	0.7071	0.6627	0.3540	0.4057	

Mean Squared Error	Weight	SVP Density	ICP Density	DCP Density	Total Retina	IRL	RPE	RNFL	IPL	INL	ORL	PRC	OPL	Number of Target Zone Crossings	% of time in sector with platform	Latency on Day 3	Latency on Day 4	Distance swam (m)	Velocity (m/s)	Thigmotaxis %	Floating %
Weight		0.000109	0.000221	0.000223	28.33047001	7.8337661	0.4083889	17.523208	14.801248	1.6035146	8.8361361	6.6275976	0.5522975	5.242241001	522.1335214	181.6059109	196.7953113	111640.432	10.805108	582.464451	56.68741
SVP Density	0.0001086		0.000248	0.000212	40.69152267	14.589362	0.426639	19.848042	15.947988	1.0933545	8.8397616	5.7669023	0.6293698	4.993716707	560.995439	178.571698	230.572394	119842.1416	12.078296	633.6155311	62.28985
ICP Density	0.0002207	0.000248		0.000266	26.60232438	9.8911133	0.336993	11.920597	12.937778	1.2222928	7.0924075	5.3114027	0.3489194	4.10402001	537.2653195	111.4725105	241.6857196	130013.5723	11.899779	623.0352754	56.975535
DCP Density	0.0002234	0.000212	0.000266		40.7837621	13.85341	0.4728084	15.486448	11.550476	1.6834773	9.6054183	6.8559301	0.6690901	4.653591305	504.0703405	116.008315	200.981531	110743.4323	11.812237	588.6869658	53.442391
Total Retina	28.33047	40.69152	26.60232	40.78376		1.6501146	0.3484704	14.44214	15.197019	1.0314674	1.6501146	1.7699878	0.3847396	4.06704223	399.5141468	180.5145242	198.2830746	110896.824	11.786889	592.9710936	57.076885
IRL	7.8337661	14.58936	9.891113	13.85341	1.650114591		0.3882219	16.630455	14.04148	0.8377632	4.8331766	3.9744671	0.4584688	3.753161458	333.7106177	184.3164611	199.0855728	110816.4459	11.732773	592.5363362	56.938432
RPE	0.4083889	0.426639	0.336993	0.472808	0.348470354	0.3882219		10.773578	14.31551	1.6945804	7.0748756	6.3370349	0.0684525	5.26508214	535.3845135	162.5184306	185.5180549	105282.017	11.775186	565.7697742	54.890654
RNFL	17.523208	19.84804	11.9206	15.48645	14.44213978	16.630455	10.773578		6.5693994	1.7564137	6.6783269	5.6474363	0.349049	5.202139641	526.4128989	128.1029067	199.3291587	111102.9297	11.596236	592.7916048	50.380598
IPL	14.801248	15.94799	12.93778	11.55048	15.19701889	14.04148	14.31551	6.5693994		1.4978586	9.8569421	7.0768017	0.6483465	5.099432739	518.7508196	150.3213503	217.4381782	111742.3735	11.810086	592.0668773	55.158252
INL	1.6035146	1.093355	1.222293	1.683477	1.031467414	0.8377632	1.6945804	1.7564137	1.4978586		7.9947991	4.9899343	0.6787405	3.22726988	263.9114308	177.5742545	217.9559385	111651.9379	11.831331	581.7611105	58.263854
ORL	8.8361361	8.839762	7.092407	9.605418	1.650114605	4.8331766	7.0748756	6.6783269	9.8569421	7.9947991		0.432734	0.3851316	4.685658183	489.9101649	176.4510124	203.2516101	111224.4127	11.838031	593.2864127	57.621778
PRC	6.6275976	5.766902	5.311403	6.85593	1.769987756	3.9744671	6.3370349	5.6474363	7.0768017	4.9899343	0.432734		0.5410623	4.580638482	484.6818465	181.750216	212.4218537	111756.6966	11.846045	590.4553656	58.430547
OPL	0.5522975	0.62937	0.348919	0.66909	0.384739607	0.4584688	0.0684525	0.349049	0.6483465	0.6787405	0.3851316	0.5410623		5.213135863	529.6888359	163.9320019	173.7966516	103918.966	11.793778	571.5077459	51.780376
Number of Target Zone Crossing	5.242241	4.993717	4.10402	4.653591	4.06704223	3.7531615	5.2650821	5.2021396	5.0994327	3.2272699	4.6856582	4.5806385	5.2131359		176.6528912	185.3112828	204.1249067	103267.6684	11.301365	473.0206523	56.516184
% of time in sector with platform	522.13352	560.9954	537.2653	504.0703	399.5141468	333.71062	535.38451	526.4129	518.75082	263.91143	489.91016	484.68185	529.68884	176.6528912		186.5650406	217.0363155	110171.2219	11.69418	530.9375604	57.138521
Latency on Day 3	181.60591	178.5717	111.4725	116.0083	180.5145242	184.31646	162.51843	128.10291	150.32135	177.57425	176.45101	181.75022	163.932	185.3112828	186.5650406		180.3057746	110544.7872	11.02726	583.2954033	48.467994
Latency on Day 4	196.79531	230.5724	241.6857	200.9815	198.2830746	199.08557	185.51805	199.32916	217.43818	217.95594	203.25161	212.42185	173.79665	204.1249067	217.0363155	180.3057746		21441.46272	8.3549825	331.8634129	17.135592
Distance swam (m)	111640.43	119842.1	130013.6	110743.4	110896.824	110816.45	105282.02	111102.93	111742.37	111651.94	111224.41	111756.7	103918.97	103267.6684	110171.2219	110544.7872	21441.46272		3.6103339	136.4316766	19.730773
Velocity (m/s)	10.805108	12.0783	11.89978	11.81224	11.78688873	11.732773	11.775186	11.596236	11.810086	11.831331	11.838031	11.846045	11.793778	11.30136511	11.69418023	11.02726003	8.354982496	3.610333879		140.2529121	37.787632
Thigmotaxis %	582.46445	633.6155	623.0353	588.687	592.9710936	592.53634	565.76977	592.7916	592.06688	581.76111	593.28641	590.45537	571.50775	473.0206523	530.9375604	583.2954033	331.8634129	136.4316766	140.25291		34.764487
Floating %	56.68741	62.28985	56.97553	53.44239	57.07688479	56.938432	54.890654	50.380598	55.158252	58.263854	57.621778	58.430547	51.780376	56.51618371	57.13852079	48.46799435	17.1355921	19.73077285	37.787632	34.76448702	

Table 4: R-squared and corresponding MSE for or all tested parameters for all transgenic animals. For the R-squared values red indicates values from 0-0.3, yellow =0.4-0.6 and green 0.6-1.

Correlation Coefficient	Weight	SVP Density	ICP Density	DCP Density	Total Retina	IRL	RPE	RNFL	IPL	INL	ORL	PRC	OPL	Number of Target Zone Crossings	% of time in sector with platform	Latency on Day 3	Latency on Day 4	Distance swam (m)	Velocity (m/s)	Thigmotaxis %	Floating %
Weight		-0.5352	0.5715	0.5770	0.6478	0.7642	0.2464	-0.1911	0.6432	0.6528	0.3549	0.2916	0.4125	0.5264	0.3967	0.3810	0.4841	0.4328	0.2766	0.2089	-0.2112
SVP Density	-0.5352		-0.7469	0.2163	-0.4732	-0.5541	-0.7167	-0.2330	-0.1754	-0.3341	-0.2659	-0.0736	-0.8647	-0.2266	-0.3171	-0.1850	0.0069	-0.0948	-0.2308	0.0176	-0.1153
ICP Density	0.5715	-0.7469		0.1723	0.4042	0.5150	0.0413	0.4730	-0.1680	0.4729	0.1519	0.0792	0.4412	0.6762	0.6046	-0.3230	0.4516	0.6341	0.6650	0.7603	0.0350
DCP Density	0.5770	0.2163	0.1723		0.0665	0.1386	-0.6039	-0.6842	0.6425	0.2913	-0.0601	0.0510	-0.4545	0.2489	0.0860	0.5218	0.5099	0.2882	-0.0537	0.2386	-0.3164
Total Retina	0.6478	-0.4732	0.4042	0.0665		0.9616	0.5047	0.0921	0.5004	0.8953	0.8977	0.8365	0.6655	0.7568	0.6931	0.0293	0.2459	0.4256	0.4775	0.3599	-0.5005
IRL	0.7642	-0.5541	0.5150	0.1386	0.9616		0.5303	0.0667	0.5472	0.9254	0.7423	0.6636	0.6576	0.7716	0.7403	-0.0103	0.1745	0.3319	0.3984	0.2855	-0.2923
RPE	0.2464	-0.7167	0.0413	-0.6039	0.5047	0.5303		0.3945	0.0358	0.2461	0.3810	0.1930	0.9031	0.0227	0.1246	-0.1941	-0.3027	-0.1001	0.2050	-0.2916	0.1554
RNFL	-0.1911	-0.2330	0.4730	-0.6842	0.0921	0.0667	0.3945		-0.7945	-0.1009	0.1178	0.0231	0.4193	0.2131	0.1912	-0.5477	0.0972	0.2914	0.4266	0.2891	0.2473
IPL	0.6432	-0.1754	-0.1680	0.6425	0.5004	0.5472	0.0358	-0.7945		0.6135	0.3432	0.3648	0.0819	0.2438	0.2382	0.4899	0.0316	-0.0496	-0.1296	-0.1185	-0.3738
INL	0.6528	-0.3341	0.4729	0.2913	0.8953	0.9254	0.2461	-0.1009	0.6135		0.7003	0.6832	0.4017	0.8660	0.8610	-0.0797	0.1166	0.2899	0.3592	0.4224	-0.3501
ORL	0.3549	-0.2659	0.1519	-0.0601	0.8977	0.7423	0.3810	0.1178	0.3432	0.7003		0.9767	0.5691	0.6091	0.5038	0.0881	0.3203	0.5062	0.5261	0.4204	-0.7526
PRC	0.2916	-0.0736	0.0792	0.0510	0.8365	0.6636	0.1930	0.0231	0.3648	0.6832	0.9767		0.3794	0.6184	0.4888	0.1098	0.3615	0.5101	0.4711	0.4581	-0.8203
OPL	0.4125	-0.8647	0.4412	-0.4545	0.6655	0.6576	0.9031	0.4193	0.0819	0.4017	0.5691	0.3794		0.2565	0.2995	-0.0407	-0.0044	0.2278	0.4633	0.0570	-0.1016
Number of Target Zone Crossings	0.5264	-0.2266	0.6762	0.2489	0.7568	0.7716	0.0227	0.2131	0.2438	0.8660	0.6091	0.6184	0.2565		0.9371	-0.1516	0.3414	0.4746	0.4227	0.6626	-0.2902
% of time in sector with platform	0.3967	-0.3171	0.6046	0.0860	0.6931	0.7403	0.1246	0.1912	0.2382	0.8610	0.5038	0.4888	0.2995	0.9371		-0.2771	0.0147	0.1914	0.2709	0.5050	-0.0793
Latency on Day 3	0.3810	-0.1850	-0.3230	0.5218	0.0293	-0.0103	-0.1941	-0.5477	0.4899	-0.0797	0.0881	0.1098	-0.0407	-0.1516	-0.2771		0.4560	0.1149	-0.2940	-0.1959	-0.4057
Latency on Day 4	0.4841	0.0069	0.4516	0.5099	0.2459	0.1745	-0.3027	0.0972	0.0316	0.1166	0.3203	0.3615	-0.0044	0.3414	0.0147	0.4560		0.8865	0.4994	0.6078	-0.5941
Distance swam (m)	0.4328	-0.0948	0.6341	0.2882	0.4256	0.3319	-0.1001	0.2914	-0.0496	0.2899	0.5062	0.5101	0.2278	0.4746	0.1914	0.1149	0.8865		0.8399	0.8298	-0.6491
Velocity (m/s)	0.2766	-0.2308	0.6650	-0.0537	0.4775	0.3984	0.2050	0.4266	-0.1296	0.3592	0.5261	0.4711	0.4633	0.4227	0.2709	-0.2940	0.4994	0.8399		0.8065	-0.4891
Thigmotaxis %	0.2089	0.0176	0.7603	0.2386	0.3599	0.2855	-0.2916	0.2891	-0.1185	0.4224	0.4204	0.4581	0.0570	0.6626	0.5050	-0.1959	0.6078	0.8298	0.8065		-0.5092
Floating %	-0.2112	-0.1153	0.0350	-0.3164	-0.5005	-0.2923	0.1554	0.2473	-0.3738	-0.3501	-0.7526	-0.8203	-0.1016	-0.2902	-0.0793	-0.4057	-0.5941	-0.6491	-0.4891	-0.5092	

P-Value	Weight	SVP Density	ICP Density	DCP Density	Total Retina	IRL	RPE	RNFL	IPL	INL	ORL	PRC	OPL	Number of Target Zone Crossings	% of time in sector with platform	Latency on Day 3	Latency on Day 4	Distance swam (m)	Velocity (m/s)	Thigmotaxis %	Floating %
Weight		0.1717	0.1801	0.1343	0.0824	0.0272	0.5563	0.6503	0.0854	0.0793	0.3884	0.4834	0.3099	0.1802	0.3306	0.3517	0.2242	0.2842	0.5073	0.6196	0.6156
SVP Density	0.1717		0.0537	0.6069	0.2363	0.1541	0.0455	0.5787	0.6779	0.4187	0.5244	0.8625	0.0056	0.5895	0.4440	0.6610	0.9871	0.8234	0.5824	0.9669	0.7857
ICP Density	0.1801	0.0537		0.7118	0.3685	0.2369	0.9300	0.2837	0.7189	0.2838	0.7451	0.8659	0.3218	0.0953	0.1504	0.4799	0.3091	0.1262	0.1031	0.0472	0.9405
DCP Density	0.1343	0.6069	0.7118		0.8756	0.7433	0.1129	0.0613	0.0858	0.4840	0.8876	0.9045	0.2579	0.5523	0.8396	0.1847	0.1968	0.4888	0.8996	0.5692	0.4452
Total Retina	0.0824	0.2363	0.3685	0.8756		0.0001	0.2021	0.8283	0.2066	0.0026	0.0025	0.0096	0.0717	0.0297	0.0566	0.9450	0.5571	0.2931	0.2315	0.3811	0.2065
IRL	0.0272	0.1541	0.2369	0.7433	0.0001		0.1764	0.8753	0.1604	0.0010	0.0349	0.0728	0.0763	0.0249	0.0357	0.9807	0.6794	0.4219	0.3283	0.4931	0.4824
RPE	0.5563	0.0455	0.9300	0.1129	0.2021	0.1764		0.3335	0.9330	0.5569	0.3518	0.6470	0.0021	0.9575	0.7687	0.6451	0.4662	0.8136	0.6263	0.4834	0.7133
RNFL	0.6503	0.5787	0.2837	0.0613	0.8283	0.8753	0.3335		0.0185	0.8121	0.7812	0.9567	0.3010	0.6123	0.6502	0.1599	0.8188	0.4838	0.2918	0.4874	0.5549
IPL	0.0854	0.6779	0.7189	0.0858	0.2066	0.1604	0.9330	0.0185		0.1057	0.4052	0.3742	0.8470	0.5607	0.5700	0.2178	0.9408	0.9071	0.7598	0.7799	0.3616
INL	0.0793	0.4187	0.2838	0.4840	0.0026	0.0010	0.5569	0.8121	0.1057		0.0531	0.0618	0.3239	0.0054	0.0060	0.8511	0.7833	0.4862	0.3822	0.2972	0.3953
ORL	0.3884	0.5244	0.7451	0.8876	0.0025	0.0349	0.3518	0.7812	0.4052	0.0531		0.0000	0.1409	0.1090	0.2030	0.8357	0.4393	0.2005	0.1804	0.2997	0.0312
PRC	0.4834	0.8625	0.8659	0.9045	0.0096	0.0728	0.6470	0.9567	0.3742	0.0618	0.0000		0.3539	0.1022	0.2191	0.7958	0.3789	0.1965	0.2386	0.2536	0.0126
OPL	0.3099	0.0056	0.3218	0.2579	0.0717	0.0763	0.0021	0.3010	0.8470	0.3239	0.1409	0.3539		0.5397	0.4711	0.9237	0.9918	0.5874	0.2476	0.8933	0.8108
Number of Target Zone Crossings	0.1802	0.5895	0.0953	0.5523	0.0297	0.0249	0.9575	0.6123	0.5607	0.0054	0.1090	0.1022	0.5397		0.0006	0.7200	0.4078	0.2347	0.2967	0.0734	0.4857
% of time in sector with platform	0.3306	0.4440	0.1504	0.8396	0.0566	0.0357	0.7687	0.6502	0.5700	0.0060	0.2030	0.2191	0.4711	0.0006		0.5064	0.9725	0.6497	0.5164	0.2017	0.8519
Latency on Day 3	0.3517	0.6610	0.4799	0.1847	0.9450	0.9807	0.6451	0.1599	0.2178	0.8511	0.8357	0.7958	0.9237	0.7200	0.5064		0.2562	0.7865	0.4797	0.6420	0.3187
Latency on Day 4	0.2242	0.9871	0.3091	0.1968	0.5571	0.6794	0.4662	0.8188	0.9408	0.7833	0.4393	0.3789	0.9918	0.4078	0.9725	0.2562		0.0033	0.2076	0.1100	0.1204
Distance swam (m)	0.2842	0.8234	0.1262	0.4888	0.2931	0.4219	0.8136	0.4838	0.9071	0.4862	0.2005	0.1965	0.5874	0.2347	0.6497	0.7865	0.0033		0.0091	0.0108	0.0816
Velocity (m/s)	0.5073	0.5824	0.1031	0.8996	0.2315	0.3283	0.6263	0.2918	0.7598	0.3822	0.1804	0.2386	0.2476	0.2967	0.5164	0.4797	0.2076	0.0091		0.0156	0.2187
Thigmotaxis %	0.6196	0.9669	0.0472	0.5692	0.3811	0.4931	0.4834	0.4874	0.7799	0.2972	0.2997	0.2536	0.8933	0.0734	0.2017	0.6420	0.1100	0.0108	0.0156		0.1974
Floating %	0.6156	0.7857	0.9405	0.4452	0.2065	0.4824	0.7133	0.5549	0.3616	0.3953	0.0312	0.0126	0.8108	0.4857	0.8519	0.3187	0.1204	0.0816	0.2187	0.1974	

Table 5: Correlation Coefficient and corresponding p-value for or all tested parameters for transgenic female animals. For the Correlation coefficient red indicates values from 0-0.3, yellow =0.3-0.5 and green 0.5-1. For the p-value red indicates values above 0.15, yellow values from 0.15-0.1 and green values from below 0.05.

R-Squared	Weight	SVP Density	ICP Density	DCP Density	Total Retina	IRL	RPE	RNFL	IPL	INL	ORL	PRC	OPL	Number of Target Zone Crossings	% of time in sector with platform	Latency on Day 3	Latency on Day 4	Distance swam (m)	Velocity (m/s)	Thigmotaxis %	Floating %
Weight		0.2864	0.3266	0.3329	0.4197	0.5840	0.0607	0.0365	0.4137	0.4262	0.1259	0.0851	0.1701	0.2771	0.1574	0.1452	0.2343	0.1873	0.0765	0.0436	0.0446
SVP Density	0.2864		0.5578	0.0468	0.2240	0.3070	0.5136	0.0543	0.0307	0.1116	0.0707	0.0054	0.7476	0.0513	0.1006	0.0342	0.0000	0.0090	0.0533	0.0003	0.0133
ICP Density	0.3266	0.5578		0.0297	0.1634	0.2652	0.0017	0.2237	0.0282	0.2236	0.0231	0.0063	0.1946	0.4573	0.3655	0.1043	0.2039	0.4021	0.4423	0.5781	0.0012
DCP Density	0.3329	0.0468	0.0297		0.0044	0.0192	0.3647	0.4681	0.4128	0.0848	0.0036	0.0026	0.2066	0.0619	0.0074	0.2723	0.2600	0.0830	0.0029	0.0570	0.1001
Total Retina	0.4197	0.2240	0.1634	0.0044		0.9246	0.2547	0.0085	0.2504	0.8016	0.8059	0.6997	0.4429	0.5728	0.4804	0.0009	0.0605	0.1811	0.2280	0.1296	0.2505
IRL	0.5840	0.3070	0.2652	0.0192	0.9246		0.2812	0.0045	0.2994	0.8564	0.5510	0.4404	0.4324	0.5954	0.5480	0.0001	0.0304	0.1102	0.1587	0.0815	0.0854
RPE	0.0607	0.5136	0.0017	0.3647	0.2547	0.2812		0.1556	0.0013	0.0605	0.1452	0.0373	0.8156	0.0005	0.0155	0.0377	0.0916	0.0100	0.0420	0.0850	0.0241
RNFL	0.0365	0.0543	0.2237	0.4681	0.0085	0.0045	0.1556		0.6312	0.0102	0.0139	0.0005	0.1759	0.0454	0.0365	0.3000	0.0095	0.0849	0.1820	0.0836	0.0612
IPL	0.4137	0.0307	0.0282	0.4128	0.2504	0.2994	0.0013	0.6312		0.3764	0.1178	0.1331	0.0067	0.0594	0.0567	0.2400	0.0010	0.0025	0.0168	0.0140	0.1397
INL	0.4262	0.1116	0.2236	0.0848	0.8016	0.8564	0.0605	0.0102	0.3764		0.4905	0.4668	0.1613	0.7499	0.7414	0.0064	0.0136	0.0840	0.1290	0.1784	0.1225
ORL	0.1259	0.0707	0.0231	0.0036	0.8059	0.5510	0.1452	0.0139	0.1178	0.4905		0.9540	0.3239	0.3710	0.2538	0.0078	0.1026	0.2562	0.2768	0.1767	0.5664
PRC	0.0851	0.0054	0.0063	0.0026	0.6997	0.4404	0.0373	0.0005	0.1331	0.4668	0.9540		0.1440	0.3824	0.2389	0.0120	0.1307	0.2602	0.2220	0.2099	0.6729
OPL	0.1701	0.7476	0.1946	0.2066	0.4429	0.4324	0.8156	0.1759	0.0067	0.1613	0.3239	0.1440		0.0658	0.0897	0.0017	0.0000	0.0519	0.2146	0.0032	0.0103
Number of Target Zone Crossings	0.2771	0.0513	0.4573	0.0619	0.5728	0.5954	0.0005	0.0454	0.0594	0.7499	0.3710	0.3824	0.0658		0.8782	0.0230	0.1166	0.2252	0.1787	0.4391	0.0842
% of time in sector with platform	0.1574	0.1006	0.3655	0.0074	0.4804	0.5480	0.0155	0.0365	0.0567	0.7414	0.2538	0.2389	0.0897	0.8782		0.0768	0.0002	0.0366	0.0734	0.2551	0.0063
Latency on Day 3	0.1452	0.0342	0.1043	0.2723	0.0009	0.0001	0.0377	0.3000	0.2400	0.0064	0.0078	0.0120	0.0017	0.0230	0.0768		0.2079	0.0132	0.0864	0.0384	0.1646
Latency on Day 4	0.2343	0.0000	0.2039	0.2600	0.0605	0.0304	0.0916	0.0095	0.0010	0.0136	0.1026	0.1307	0.0000	0.1166	0.0002	0.2079		0.7860	0.2494	0.3694	0.3529
Distance swam (m)	0.1873	0.0090	0.4021	0.0830	0.1811	0.1102	0.0100	0.0849	0.0025	0.0840	0.2562	0.2602	0.0519	0.2252	0.0366	0.0132	0.7860		0.7055	0.6885	0.4213
Velocity (m/s)	0.0765	0.0533	0.4423	0.0029	0.2280	0.1587	0.0420	0.1820	0.0168	0.1290	0.2768	0.2220	0.2146	0.1787	0.0734	0.0864	0.2494	0.7055		0.6504	0.2392
Thigmotaxis %	0.0436	0.0003	0.5781	0.0570	0.1296	0.0815	0.0850	0.0836	0.0140	0.1784	0.1767	0.2099	0.0032	0.4391	0.2551	0.0384	0.3694	0.6885	0.6504		0.2593
Floating %	0.0446	0.0133	0.0012	0.1001	0.2505	0.0854	0.0241	0.0612	0.1397	0.1225	0.5664	0.6729	0.0103	0.0842	0.0063	0.1646	0.3529	0.4213	0.2392	0.2593	

Mean Squared Error	Weight	SVP Density	ICP Density	DCP Density	Total Retina	IRL	RPE	RNFL	IPL	INL	ORL	PRC	OPL	Number of Target Zone Crossings	% of time in sector with platform	Latency on Day 3	Latency on Day 4	Distance swam (m)	Velocity (m/s)	Thigmotaxis %	Floating %
Weight		2.5E-05	0.00015	0.00015	20.44	6.33229	0.10762	16.2326	12.91629	0.752343	5.16847	4.27288	0.2349	4.834440228	663.696694	138.840028	79.4472081	55822.08828	6.874115	517.4121637	30.022995
SVP Density	2.52E-05		9.9E-05	0.00022	27.33383	10.5499	0.05572	15.9331	21.35128	1.164792	5.49483	4.64481	0.07143	6.344224871	708.4117567	156.863957	103.753507	68069.60648	7.047	540.8494704	31.007236
ICP Density	0.000151	9.9E-05		0.00025	24.97269	9.49204	0.08741	14.4528	18.46218	1.020539	5.40026	4.85852	0.12199	4.097966629	550.4214534	120.405659	94.3608178	46933.0212	4.738972	254.1960718	34.221001
DCP Density	0.000151	0.00022	0.00025		35.06596	14.931	0.07279	8.96152	12.93424	1.199863	5.89168	4.65796	0.22459	6.273354004	781.8119765	118.198407	76.7863436	62982.25457	7.422007	510.2066824	28.279017
Total Retina	20.44	27.3338	24.9727	35.066		1.14758	0.08539	16.7048	16.51306	0.260099	1.14758	1.40245	0.15769	2.856941366	409.2282346	162.281898	97.4824336	56243.87753	5.746362	470.9243754	23.55293
IRL	6.332288	10.5499	9.49204	14.931	1.14758		0.08236	16.7727	15.43253	0.188337	2.65507	2.61339	0.16065	2.705921384	355.9805403	162.404517	100.599445	61119.45683	6.262087	496.9173903	28.740839
RPE	0.107617	0.05572	0.08741	0.07279	0.085389	0.08236		14.2259	22.0004	1.231716	5.05472	4.49609	0.05221	6.684059693	775.4015023	156.30116	94.2532587	67998.18084	7.130734	495.0114719	30.666276
RNFL	16.23255	15.9331	14.4528	8.96152	16.70475	16.7727	14.2259		8.124881	1.297763	5.83102	4.66762	0.23328	6.383710706	758.8527523	113.697651	102.777549	62854.42543	6.08861	495.8126219	29.503177
IPL	12.91629	21.3513	18.4622	12.9342	16.51306	15.4325	22.0004	8.12488		0.817556	5.21646	4.04854	0.28116	6.290025214	742.9512242	123.440902	103.654941	68517.23901	7.318519	533.4222064	27.033446
INL	0.752343	1.16479	1.02054	1.19986	0.260099	0.18834	1.23172	1.29776	0.817556		3.01296	2.49026	0.23739	1.672713718	203.717232	161.389098	102.347512	62915.44673	6.483088	444.5053991	27.574258
ORL	5.168471	5.49483	5.40026	5.89168	1.14758	2.65507	5.05472	5.83102	5.216462	3.012961		0.21503	0.19137	4.20676334	587.6953119	161.161121	93.1152807	51086.40764	5.382901	445.4122402	13.625804
PRC	4.272876	4.64481	4.85852	4.65796	1.402449	2.61339	4.49609	4.66762	4.048542	2.490264	0.21503		0.24231	4.130184751	599.4770359	160.464892	90.1973081	50811.68071	5.791254	427.4618796	10.277708
OPL	0.234902	0.07143	0.12199	0.22459	0.157687	0.16065	0.05221	0.23328	0.281159	0.237389	0.19137	0.24231		6.247431646	716.972355	162.152076	103.756411	65121.00457	5.845865	539.259629	31.100617
Number of Target Zone Crossings	4.83444	6.34422	4.09797	6.27335	2.856941	2.70592	6.68406	6.38371	6.290025	1.672714	4.20676	4.13018	6.24743		95.97227527	158.687267	91.662074	53216.20793	6.113215	303.4833116	28.778599
% of time in sector with platform	663.6967	708.412	550.421	781.812	409.2282	355.981	775.402	758.853	742.9512	203.7172	587.695	599.477	716.972	95.97227527		149.95037	103.736116	66169.38227	6.897307	403.0187987	31.227192
Latency on Day 3	138.84	156.864	120.406	118.198	162.2819	162.405	156.301	113.698	123.4409	161.3891	161.161	160.465	162.152	158.6872667	149.9503703		82.1862292	67779.95328	6.800146	520.2629277	26.253141
Latency on Day 4	79.44721	103.754	94.3608	76.7863	97.48243	100.599	94.2533	102.778	103.6549	102.3475	93.1153	90.1973	103.756	91.66207398	103.7361156	82.1862292		14700.96181	5.586709	341.1856838	20.334319
Distance swam (m)	55822.09	68069.6	46933	62982.3	56243.88	61119.5	67998.2	62854.4	68517.24	62915.45	51086.4	50811.7	65121	53216.20793	66169.38227	67779.9533	14700.9618		2.192179	168.5024331	18.184275
Velocity (m/s)	6.874115	7.047	4.73897	7.42201	5.746362	6.26209	7.13073	6.08861	7.318519	6.483088	5.3829	5.79125	5.84586	6.11321548	6.897306998	6.80014582	5.58670878	2.192179392		189.1522381	23.906569
Thigmotaxis %	517.4122	540.849	254.196	510.207	470.9244	496.917	495.011	495.813	533.4222	444.5054	445.412	427.462	539.26	303.4833116	403.0187987	520.262928	341.185684	168.5024331	189.1522		23.276919
Floating %	30.023	31.0072	34.221	28.279	23.55293	28.7408	30.6663	29.5032	27.03345	27.57426	13.6258	10.2777	31.1006	28.77859921	31.22719228	26.2531411	20.3343194	18.1842748	23.90657	23.27691934	

Table 6: R-squared and corresponding MSE for or all tested parameters for transgenic female animals. For the R-squared values red indicates values from 0-0.3, yellow =0.4-0.6 and green 0.6-1.

Correlation Coefficient	Weight	SVP Density	ICP Density	DCP Density	Total Retina	IRL	RPE	RNFL	IPL	INL	ORL	PRC	OPL	Number of Target Zone Crossings	% of time in sector with platform	Latency on Day 3	Latency on Day 4	Distance swam (m)	Velocity (m/s)	Thigmotaxis %	Floating %
Weight		0.9647	-0.4067	0.5965	0.0326	0.4723	-0.3850	-0.3904	0.6394	0.6159	-0.2827	-0.2279	-0.3112	0.5181	0.5155	0.7656	-0.1823	-0.2081	-0.1187	-0.0557	0.0752
SVP Density	0.9647		-0.6232	0.6485	-0.4845	0.2209	-0.4744	-0.6945	0.8426	0.7571	-0.7987	-0.8585	-0.4867	0.2974	0.5438	0.8488	0.0277	-0.0233	0.0591	0.0822	-0.1988
ICP Density	-0.4067	-0.6232		-0.1271	0.5943	0.1868	0.7312	0.7280	-0.5855	-0.8241	0.6971	0.5460	0.8340	0.3921	-0.4815	-0.6633	-0.4138	-0.3699	-0.2085	-0.3128	0.5402
DCP Density	0.5965	0.6485	-0.1271		-0.0410	0.2447	0.3480	-0.0164	0.1853	0.1276	-0.2373	-0.3515	0.2403	0.4879	0.7163	0.7737	0.0411	-0.0864	0.0314	-0.0572	-0.2498
Total Retina	0.0326	-0.4845	0.5943	-0.0410		0.8849	0.3722	0.8289	-0.6481	0.5573	0.9443	0.9248	0.5277	0.4142	0.3342	0.0103	-0.5052	-0.5152	-0.6389	-0.5411	0.5608
IRL	0.4723	0.2209	0.1868	0.2447	0.8849		0.1553	0.5426	-0.2570	0.7656	0.6823	0.6861	0.3251	0.4957	0.5448	0.3818	-0.5866	-0.6184	-0.7014	-0.5919	0.5590
RPE	-0.3850	-0.4744	0.7312	0.3480	0.3722	0.1553		0.7836	-0.7025	-0.4165	0.4746	0.2444	0.9548	0.2263	0.1053	-0.1922	-0.2113	-0.2872	-0.2084	-0.3589	0.1652
RNFL	-0.3904	-0.6945	0.7280	-0.0164	0.8289	0.5426	0.7836		-0.9139	0.0476	0.9177	0.7927	0.8445	0.2858	0.1550	-0.2609	-0.3512	-0.3836	-0.4537	-0.4669	0.4018
IPL	0.6394	0.8426	-0.5855	0.1853	-0.6481	-0.2570	-0.7025	-0.9139		0.0704	-0.8357	-0.7510	-0.6783	-0.1509	-0.0855	0.3657	-0.0049	0.0187	0.1465	0.1501	-0.0860
INL	0.6159	0.7571	-0.8241	0.1276	0.5573	0.7656	-0.4165	0.0476	0.0704		0.3338	0.4903	-0.3249	0.3791	0.6529	0.6175	-0.1198	-0.1344	-0.3131	-0.1136	0.1069
ORL	-0.2827	-0.7987	0.6971	-0.2373	0.9443	0.6823	0.4746	0.9177	-0.8357	0.3338		0.9667	0.5985	0.2998	0.1395	-0.2537	-0.3784	-0.3716	-0.5072	-0.4311	0.4852
PRC	-0.2279	-0.8585	0.5460	-0.3515	0.9248	0.6861	0.2444	0.7927	-0.7510	0.4903	0.9667		0.3735	0.2632	0.1606	-0.2038	-0.2913	-0.2680	-0.4552	-0.3262	0.4205
OPL	-0.3112	-0.4867	0.8340	0.2403	0.5277	0.3251	0.9548	0.8445	-0.6783	-0.3249	0.5985	0.3735		0.2628	0.0029	-0.2815	-0.4596	-0.5079	-0.4133	-0.5414	0.4424
Number of Target Zone Crossings	0.5181	0.2974	0.3921	0.4879	0.4142	0.4957	0.2263	0.2858	-0.1509	0.3791	0.2998	0.2632	0.2628		0.4235	0.3377	0.0582	0.0533	0.1648	0.1935	-0.0067
% of time in sector with platform	0.5155	0.5438	-0.4815	0.7163	0.3342	0.5448	0.1053	0.1550	-0.0855	0.6529	0.1395	0.1606	0.0029	0.4235		0.8954	0.1668	0.0403	-0.0850	-0.0222	-0.3268
Latency on Day 3	0.7656	0.8488	-0.6633	0.7737	0.0103	0.3818	-0.1922	-0.2609	0.3657	0.6175	-0.2537	-0.2038	-0.2815	0.3377	0.8954		0.1582	0.0484	-0.0027	0.0500	-0.3515
Latency on Day 4	-0.1823	0.0277	-0.4138	0.0411	-0.5052	-0.5866	-0.2113	-0.3512	-0.0049	-0.1198	-0.3784	-0.2913	-0.4596	0.0582	0.1668	0.1582		0.9865	0.9190	0.9336	-0.9608
Distance swam (m)	-0.2081	-0.0233	-0.3699	-0.0864	-0.5152	-0.6184	-0.2872	-0.3836	0.0187	-0.1344	-0.3716	-0.2680	-0.5079	0.0533	0.0403	0.0484	0.9865		0.9420	0.9687	-0.9080
Velocity (m/s)	-0.1187	0.0591	-0.2085	0.0314	-0.6389	-0.7014	-0.2084	-0.4537	0.1465	-0.3131	-0.5072	-0.4552	-0.4133	0.1648	-0.0850	-0.0027	0.9190	0.9420		0.9724	-0.8444
Thigmotaxis %	-0.0557	0.0822	-0.3128	-0.0572	-0.5411	-0.5919	-0.3589	-0.4669	0.1501	-0.1136	-0.4311	-0.3262	-0.5414	0.1935	-0.0222	0.0500	0.9336	0.9687	0.9724		-0.8351
Floating %	0.0752	-0.1988	0.5402	-0.2498	0.5608	0.5590	0.1652	0.4018	-0.0860	0.1069	0.4852	0.4205	0.4424	-0.0067	-0.3268	-0.3515	-0.9608	-0.9080	-0.8444	-0.8351	

P-Value	Weight	SVP Density	ICP Density	DCP Density	Total Retina	IRL	RPE	RNFL	IPL	INL	ORL	PRC	OPL	Number of Target Zone Crossings	% of time in sector with platform	Latency on Day 3	Latency on Day 4	Distance swam (m)	Velocity (m/s)	Thigmotaxis %	Floating %
Weight		0.0079	0.4969	0.2114	0.9512	0.3442	0.4511	0.4441	0.1716	0.1930	0.5873	0.6641	0.5483	0.2924	0.2952	0.0759	0.7296	0.6923	0.8228	0.9165	0.8874
SVP Density	0.0079		0.2614	0.2365	0.4082	0.7211	0.4195	0.1931	0.0732	0.1383	0.1051	0.0625	0.4057	0.6270	0.3434	0.0689	0.9647	0.9703	0.9248	0.8954	0.7486
ICP Density	0.4969	0.2614		0.8386	0.2906	0.7636	0.1604	0.1631	0.2996	0.0862	0.1907	0.3411	0.0792	0.5139	0.4115	0.2223	0.4886	0.5400	0.7365	0.6084	0.3473
DCP Density	0.2114	0.2365	0.8386		0.9385	0.6403	0.4991	0.9754	0.7252	0.8097	0.6508	0.4944	0.6465	0.3263	0.1093	0.0710	0.9384	0.8707	0.9529	0.9143	0.6331
Total Retina	0.9512	0.4082	0.2906	0.9385		0.0191	0.4674	0.0414	0.1639	0.2506	0.0046	0.0083	0.2820	0.4142	0.5174	0.9846	0.3067	0.2956	0.1720	0.2675	0.2470
IRL	0.3442	0.7211	0.7636	0.6403	0.0191		0.7690	0.2659	0.6230	0.0760	0.1354	0.1323	0.5296	0.3173	0.2636	0.4552	0.2210	0.1906	0.1205	0.2158	0.2488
RPE	0.4511	0.4195	0.1604	0.4991	0.4674	0.7690		0.0652	0.1196	0.4114	0.3416	0.6407	0.0030	0.6664	0.8427	0.7153	0.6878	0.5811	0.6919	0.4848	0.7545
RNFL	0.4441	0.1931	0.1631	0.9754	0.0414	0.2659	0.0652		0.0108	0.9286	0.0099	0.0600	0.0344	0.5830	0.7694	0.6175	0.4948	0.4528	0.3661	0.3505	0.4298
IPL	0.1716	0.0732	0.2996	0.7252	0.1639	0.6230	0.1196	0.0108		0.8946	0.0383	0.0853	0.1386	0.7753	0.8721	0.4759	0.9927	0.9719	0.7819	0.7765	0.8713
INL	0.1930	0.1383	0.0862	0.8097	0.2506	0.0760	0.4114	0.9286	0.8946		0.5179	0.3234	0.5298	0.4586	0.1598	0.1915	0.8211	0.7996	0.5457	0.8303	0.8403
ORL	0.5873	0.1051	0.1907	0.6508	0.0046	0.1354	0.3416	0.0099	0.0383	0.5179		0.0016	0.2094	0.5638	0.7921	0.6277	0.4595	0.4682	0.3045	0.3934	0.3293
PRC	0.6641	0.0625	0.3411	0.4944	0.0083	0.1323	0.6407	0.0600	0.0853	0.3234	0.0016		0.4658	0.6143	0.7611	0.6986	0.5754	0.6076	0.3643	0.5281	0.4064
OPL	0.5483	0.4057	0.0792	0.6465	0.2820	0.5296	0.0030	0.0344	0.1386	0.5298	0.2094	0.4658		0.6149	0.9957	0.5889	0.3591	0.3037	0.4153	0.2673	0.3796
Number of Target Zone Crossings	0.2924	0.6270	0.5139	0.3263	0.4142	0.3173	0.6664	0.5830	0.7753	0.4586	0.5638	0.6143	0.6149		0.4027	0.5127	0.9128	0.9201	0.7550	0.7134	0.9899
% of time in sector with platform	0.2952	0.3434	0.4115	0.1093	0.5174	0.2636	0.8427	0.7694	0.8721	0.1598	0.7921	0.7611	0.9957	0.4027		0.0158	0.7521	0.9396	0.8727	0.9666	0.5273
Latency on Day 3	0.0759	0.0689	0.2223	0.0710	0.9846	0.4552	0.7153	0.6175	0.4759	0.1915	0.6277	0.6986	0.5889	0.5127	0.0158		0.7646	0.9275	0.9960	0.9250	0.4944
Latency on Day 4	0.7296	0.9647	0.4886	0.9384	0.3067	0.2210	0.6878	0.4948	0.9927	0.8211	0.4595	0.5754	0.3591	0.9128	0.7521	0.7646		0.0003	0.0096	0.0065	0.0023
Distance swam (m)	0.6923	0.9703	0.5400	0.8707	0.2956	0.1906	0.5811	0.4528	0.9719	0.7996	0.4682	0.6076	0.3037	0.9201	0.9396	0.9275	0.0003		0.0049	0.0014	0.0123
Velocity (m/s)	0.8228	0.9248	0.7365	0.9529	0.1720	0.1205	0.6919	0.3661	0.7819	0.5457	0.3045	0.3643	0.4153	0.7550	0.8727	0.9960	0.0096	0.0049		0.0011	0.0344
Thigmotaxis %	0.9165	0.8954	0.6084	0.9143	0.2675	0.2158	0.4848	0.3505	0.7765	0.8303	0.3934	0.5281	0.2673	0.7134	0.9666	0.9250	0.0065	0.0014	0.0011		0.0385
Floating %	0.8874	0.7486	0.3473	0.6331	0.2470	0.2488	0.7545	0.4298	0.8713	0.8403	0.3293	0.4064	0.3796	0.9899	0.5273	0.4944	0.0023	0.0123	0.0344	0.0385	

Table 7: Correlation Coefficient and corresponding p-value for or all tested parameters for transgenic male animals. For the Correlation coefficient red indicates values from 0-0.3, yellow =0.3-0.5 and green 0.5-1. For the p-value red indicates values above 0.15. yellow values from 0.15-0.1 and green values from below 0.05.

R-Squared	Weight	SVP Density	ICP Density	DCP Density	Total Retina	IRL	RPE	RNFL	IPL	INL	ORL	PRC	OPL	Number of Target Zone Crossings	% of time in sector with platform	Latency on Day 3	Latency on Day 4	Distance swam (m)	Velocity (m/s)	Thigmotaxis %	Floating %
Weight		0.9306	0.1654	0.3558	0.0011	0.2231	0.1482	0.1524	0.4088	0.3793	0.0799	0.0519	0.0968	0.2684	0.2658	0.5862	0.0332	0.0433	0.0141	0.0031	0.0057
SVP Density	0.9306		0.3884	0.4205	0.2347	0.0488	0.2251	0.4824	0.7099	0.5732	0.6380	0.7370	0.2369	0.0884	0.2958	0.7205	0.0008	0.0005	0.0035	0.0068	0.0395
ICP Density	0.1654	0.3884		0.0162	0.3531	0.0349	0.5346	0.5300	0.3428	0.6792	0.4860	0.2981	0.6955	0.1537	0.2318	0.4400	0.1712	0.1368	0.0435	0.0978	0.2918
DCP Density	0.3558	0.4205	0.0162		0.0017	0.0599	0.1211	0.0003	0.0344	0.0163	0.0563	0.1236	0.0578	0.2380	0.5131	0.5987	0.0017	0.0075	0.0010	0.0033	0.0624
Total Retina	0.0011	0.2347	0.3531	0.0017		0.7831	0.1386	0.6871	0.4201	0.3106	0.8916	0.8552	0.2784	0.1716	0.1117	0.0001	0.2552	0.2654	0.4082	0.2928	0.3145
IRL	0.2231	0.0488	0.0349	0.0599	0.7831		0.0241	0.2945	0.0660	0.5861	0.4655	0.4708	0.1057	0.2458	0.2968	0.1457	0.3441	0.3824	0.4919	0.3504	0.3125
RPE	0.1482	0.2251	0.5346	0.1211	0.1386	0.0241		0.6140	0.4936	0.1735	0.2252	0.0597	0.9117	0.0512	0.0111	0.0369	0.0446	0.0825	0.0434	0.1288	0.0273
RNFL	0.1524	0.4824	0.5300	0.0003	0.6871	0.2945	0.6140		0.8352	0.0023	0.8421	0.6284	0.7132	0.0817	0.0240	0.0681	0.1233	0.1471	0.2059	0.2180	0.1614
IPL	0.4088	0.7099	0.3428	0.0344	0.4201	0.0660	0.4936	0.8352		0.0050	0.6984	0.5639	0.4601	0.0228	0.0073	0.1338	0.0000	0.0004	0.0215	0.0225	0.0074
INL	0.3793	0.5732	0.6792	0.0163	0.3106	0.5861	0.1735	0.0023	0.0050		0.1114	0.2404	0.1056	0.1437	0.4263	0.3813	0.0144	0.0181	0.0980	0.0129	0.0114
ORL	0.0799	0.6380	0.4860	0.0563	0.8916	0.4655	0.2252	0.8421	0.6984	0.1114		0.9345	0.3582	0.0899	0.0195	0.0643	0.1432	0.1381	0.2572	0.1858	0.2355
PRC	0.0519	0.7370	0.2981	0.1236	0.8552	0.4708	0.0597	0.6284	0.5639	0.2404	0.9345		0.1395	0.0693	0.0258	0.0415	0.0849	0.0718	0.2072	0.1064	0.1768
OPL	0.0968	0.2369	0.6955	0.0578	0.2784	0.1057	0.9117	0.7132	0.4601	0.1056	0.3582	0.1395		0.0690	0.0000	0.0792	0.2113	0.2580	0.1708	0.2931	0.1958
Number of Target Zone Crossings	0.2684	0.0884	0.1537	0.2380	0.1716	0.2458	0.0512	0.0817	0.0228	0.1437	0.0899	0.0693	0.0690		0.1793	0.1140	0.0034	0.0028	0.0272	0.0374	0.0000
% of time in sector with platform	0.2658	0.2958	0.2318	0.5131	0.1117	0.2968	0.0111	0.0240	0.0073	0.4263	0.0195	0.0258	0.0000	0.1793		0.8017	0.0278	0.0016	0.0072	0.0005	0.1068
Latency on Day 3	0.5862	0.7205	0.4400	0.5987	0.0001	0.1457	0.0369	0.0681	0.1338	0.3813	0.0643	0.0415	0.0792	0.1140	0.8017		0.0250	0.0023	0.0000	0.0025	0.1236
Latency on Day 4	0.0332	0.0008	0.1712	0.0017	0.2552	0.3441	0.0446	0.1233	0.0000	0.0144	0.1432	0.0849	0.2113	0.0034	0.0278	0.0250		0.9732	0.8446	0.8716	0.9231
Distance swam (m)	0.0433	0.0005	0.1368	0.0075	0.2654	0.3824	0.0825	0.1471	0.0004	0.0181	0.1381	0.0718	0.2580	0.0028	0.0016	0.0023	0.9732		0.8874	0.9385	0.8245
Velocity (m/s)	0.0141	0.0035	0.0435	0.0010	0.4082	0.4919	0.0434	0.2059	0.0215	0.0980	0.2572	0.2072	0.1708	0.0272	0.0072	0.0000	0.8446	0.8874		0.9456	0.7130
Thigmotaxis %	0.0031	0.0068	0.0978	0.0033	0.2928	0.3504	0.1288	0.2180	0.0225	0.0129	0.1858	0.1064	0.2931	0.0374	0.0005	0.0025	0.8716	0.9385	0.9456		0.6974
Floating %	0.0057	0.0395	0.2918	0.0624	0.3145	0.3125	0.0273	0.1614	0.0074	0.0114	0.2355	0.1768	0.1958	0.0000	0.1068	0.1236	0.9231	0.8245	0.7130	0.6974	

Mean Squared Error	Weight	SVP Density	ICP Density	DCP Density	Total Retina	IRL	RPE	RNFL	IPL	INL	ORL	PRC	OPL	Number of Target Zone Crossings	% of time in sector with platform	Latency on Day 3	Latency on Day 4	Distance swam (m)	Velocity (m/s)	Thigmotaxis %	Floating %
Weight		1.86E-05	0.0001814	0.0001418	29.675997	4.67889	0.57557	10.6256	3.68647	1.46096	11.0936	8.52552	0.73806	2.113372093	148.5595	53.7531001	277.082605	160362.109	14.94103	644.8711687	87.1992
SVP Density	1.86E-05		0.0001329	0.00015148	12.070748	3.05926	0.60711	6.76741	1.98446	0.36865	3.05505	1.16954	0.74562	1.96895531	143.6274541	41.4134741	341.79219	200736.477	17.54716	770.8909933	97.2778
ICP Density	0.000181	0.000133		0.00025719	10.202998	3.10396	0.36462	6.1447	4.49588	0.27712	4.33778	3.12139	0.29754	1.827987981	156.6650566	82.984163	283.490503	173361.784	16.84326	700.2202774	71.7211
DCP Density	0.000142	0.000151	0.0002572		29.657571	5.66201	0.59388	12.5333	6.02154	2.31553	11.3783	7.88127	0.76999	2.201339193	98.5180404	52.1375152	286.124339	166371.285	15.13955	644.7653171	82.2229
Total Retina	29.676	12.07075	10.202998	29.6575714		1.30648	0.58209	3.92219	3.61628	1.62266	1.30648	1.30189	0.58966	2.393276457	179.7347383	129.892608	213.458238	123132.714	8.968528	457.456661	60.1131
IRL	4.678894	3.05926	3.1039553	5.66201169	1.3064792		0.65943	8.8452	5.8239	0.97431	6.44454	4.75888	0.73083	2.178935698	142.2770154	110.974347	187.984069	103519.511	7.700075	420.2360444	60.2932
RPE	0.575567	0.607106	0.3646172	0.59388267	0.5820928	0.65943		4.83937	3.15797	1.94548	9.34182	8.45545	0.07216	2.741004972	200.0895209	125.108555	273.812468	153798.782	14.49617	563.5627017	85.3031
RNFL	10.62555	6.767406	6.1447009	12.533276	3.9221925	8.8452	4.83937		1.02757	2.34849	1.90376	3.3414	0.23439	2.65294271	197.4717367	121.063281	251.255589	142959.446	12.03451	505.8657561	73.5405
IPL	3.68647	1.984461	4.4958789	6.02153635	3.6162756	5.8239	3.15797	1.02757		2.34216	3.636	3.92129	0.4412	2.82306419	200.8532532	112.529389	286.601204	167564.831	14.8294	632.3029515	87.0469
INL	1.46096	0.368649	0.2771236	2.31552883	1.6226644	0.97431	1.94548	2.34849	2.34216		10.7138	6.83052	0.73091	2.473701295	116.0822197	80.3759017	282.492828	164594.753	13.6689	638.5284435	86.6931
ORL	11.09362	3.055052	4.3377794	11.3783118	1.3064793	6.44454	9.34182	1.90376	3.636	10.7138		0.58929	0.52447	2.629246299	198.392309	121.547922	245.56624	144474.11	11.25616	526.6759691	67.0461
PRC	8.525522	1.169543	3.1213921	7.88126787	1.3018927	4.75888	8.45545	3.3414	3.92129	6.83052	0.58929		0.7032	2.688798348	197.110215	124.512447	262.28635	155582.686	12.01415	578.0664446	72.1889
OPL	0.738057	0.745616	0.2975387	0.76999256	0.5896645	0.73083	0.07216	0.23439	0.4412	0.73091	0.52447	0.7032		2.689434791	202.3293902	119.612939	226.060635	124382.756	12.56574	457.2888631	70.5284
Number of Target Zone Crossings	2.113372	1.968955	1.827988	2.20133919	2.3932765	2.17894	2.741	2.65294	2.82306	2.4737	2.62925	2.6888	2.68943		166.0452846	115.093324	285.63714	167147.277	14.74294	622.6715478	87.6912
% of time in sector with platform	148.5595	143.6275	156.66506	98.5180404	179.73474	142.277	200.09	197.472	200.853	116.082	198.392	197.11	202.329	166.0452846		25.7624278	278.634773	167351.38	15.04489	646.5601852	78.3311
Latency on Day 3	53.7531	41.41347	82.984163	52.1375152	129.89261	110.974	125.109	121.063	112.529	80.3759	121.548	124.512	119.613	115.0933241	25.76242782		279.432413	167231.47	15.15438	645.2622457	76.8585
Latency on Day 4	277.0826	341.7922	283.4905	286.124339	213.45824	187.984	273.812	251.256	286.601	282.493	245.566	262.286	226.061	285.6371396	278.6347729	279.432413		4493.33792	2.354646	83.05890433	6.74114
Distance swam (m)	160362.1	200736.5	173361.78	166371.285	123132.71	103520	153799	142959	167565	164595	144474	155583	124383	167147.2765	167351.3802	167231.47	4493.33792		1.706364	39.80308986	15.3945
Velocity (m/s)	14.94103	17.54716	16.843255	15.13955	8.9685275	7.70007	14.4962	12.0345	14.8294	13.6689	11.2562	12.0141	12.5657	14.74294464	15.04488511	15.15438	2.35464646	1.70636392		35.21030704	25.1707
Thigmotaxis %	644.8712	770.891	700.22028	644.765317	457.45666	420.236	563.563	505.866	632.303	638.528	526.676	578.066	457.289	622.6715478	646.5601852	645.262246	83.0589043	39.8030899	35.21031		26.5353
Floating %	87.1992	97.27783	71.721079	82.2229083	60.113118	60.2932	85.3031	73.5405	87.0469	86.6931	67.0461	72.1889	70.5284	87.69124506	78.33110984	76.8585355	6.74114249	15.3944811	25.1707	26.53533702	

Table 8: R-squared and corresponding MSE for or all tested parameters for transgenic male animals. For the R-squared values red indicates values from 0-0.3, yellow =0.4-0.6 and green 0.6-1.

Correlation Coefficient	Weight	SVP Density	ICP Density	DCP Density	Total Retina	IRL	RPE	RNFL	IPL	INL	ORL	PRC	OPL	Number of Target Zone Crossings	% of time in sector with platform	Latency on Day 3	Latency on Day 4	Distance swam (m)	Velocity (m/s)	Thigmotaxis %	Floating %
Weight		-0.3831	-0.5340	0.0413	0.1072	0.1121	-0.0039	0.1159	-0.0147	-0.0704	0.0925	0.0465	0.2940	0.1706	0.0857	-0.3058	0.4759	0.2756	-0.2061	0.4310	0.1287
SVP Density	-0.3831		0.1815	0.0434	0.0866	0.2714	0.2938	0.1296	0.0984	0.0465	-0.0590	-0.0872	0.1353	0.1617	-0.0057	-0.2025	-0.1877	0.1667	0.5825	-0.0843	-0.0599
ICP Density	-0.5340	0.1815		0.4147	-0.0548	0.0730	-0.0416	-0.1645	0.1633	0.3737	-0.1436	-0.0655	-0.4934	-0.3925	-0.5363	0.2955	-0.3655	-0.2691	0.1663	-0.4188	-0.0170
DCP Density	0.0413	0.0434	0.4147		0.4611	0.5228	0.2156	0.2735	-0.0844	0.5307	0.3676	0.3873	0.0317	-0.2399	-0.2850	0.2752	-0.1121	0.0897	0.3523	-0.2443	-0.3839
Total Retina	0.1072	0.0866	-0.0548	0.4611		0.9183	0.5124	0.8852	-0.6970	0.7088	0.9563	0.9412	0.4546	-0.3674	-0.3715	0.0340	0.0041	-0.0350	-0.1097	-0.2272	-0.0200
IRL	0.1121	0.2714	0.0730	0.5228	0.9183		0.5685	0.8547	-0.5797	0.7732	0.7624	0.7461	0.3860	-0.3093	-0.4169	0.1028	0.0605	-0.0266	-0.1175	-0.0994	0.0268
RPE	-0.0039	0.2938	-0.0416	0.2156	0.5124	0.5685		0.7362	-0.5423	0.0632	0.4176	0.3033	0.8027	-0.5218	-0.5653	0.0265	0.3210	0.3660	0.0960	0.1963	-0.2810
RNFL	0.1159	0.1296	-0.1645	0.2735	0.8852	0.8547	0.7362		-0.8845	0.5565	0.8155	0.7661	0.5927	-0.4615	-0.4508	0.0660	0.2554	0.1002	-0.2650	-0.0162	0.0952
IPL	-0.0147	0.0984	0.1633	-0.0844	-0.6970	#####	-0.5423	-0.8845		-0.4899	-0.7112	-0.6911	-0.3879	0.5236	0.3813	-0.0943	-0.2372	0.0076	0.4621	0.1244	-0.2338
INL	-0.0704	0.0465	0.3737	0.5307	0.7088	0.7732	0.0632	0.5565	-0.4899		0.5874	0.6651	-0.2080	-0.2753	-0.2920	0.2153	-0.2106	-0.3955	-0.3260	-0.4231	0.2108
ORL	0.0925	-0.0590	-0.1436	0.3676	0.9563	0.7624	0.4176	0.8155	-0.7112	0.5874		0.9874	0.4579	-0.3721	-0.2993	-0.0217	-0.0400	-0.0392	-0.0966	-0.3120	-0.0551
PRC	0.0465	-0.0872	-0.0655	0.3873	0.9412	0.7461	0.3033	0.7661	-0.6911	0.6651	0.9874		0.3112	-0.3362	-0.2688	-0.0078	-0.1361	-0.1609	-0.1444	-0.4084	0.0226
OPL	0.2940	0.1353	-0.4934	0.0317	0.4546	0.3860	0.8027	0.5927	-0.3879	-0.2080	0.4579	0.3112		-0.3452	-0.2871	-0.0819	0.4950	0.6312	0.2176	0.3947	-0.4336
Number of Target Zone Crossings	0.1706	0.1617	-0.3925	-0.2399	-0.3674	#####	-0.5218	-0.4615	0.5236	-0.2753	-0.3721	-0.3362	-0.3452		0.8461	-0.4649	-0.3560	-0.3593	0.0193	0.0159	0.2382
% of time in sector with platform	0.0857	-0.0057	-0.5363	-0.2850	-0.3715	#####	-0.5653	-0.4508	0.3813	-0.2920	-0.2993	-0.2688	-0.2871	0.8461		-0.3971	-0.3912	-0.3051	0.0829	-0.1560	-0.0158
Latency on Day 3	-0.3058	-0.2025	0.2955	0.2752	0.0340	0.1028	0.0265	0.0660	-0.0943	0.2153	-0.0217	-0.0078	-0.0819	-0.4649	-0.3971		0.4265	0.3401	-0.0829	0.3573	-0.0443
Latency on Day 4	0.4759	-0.1877	-0.3655	-0.1121	0.0041	0.0605	0.3210	0.2554	-0.2372	-0.2106	-0.0400	-0.1361	0.4950	-0.3560	-0.3912	0.4265		0.7887	-0.2114	0.8487	0.0952
Distance swam (m)	0.2756	0.1667	-0.2691	0.0897	-0.0350	#####	0.3660	0.1002	0.0076	-0.3955	-0.0392	-0.1609	0.6312	-0.3593	-0.3051	0.3401	0.7887		0.4166	0.6472	-0.3781
Velocity (m/s)	-0.2061	0.5825	0.1663	0.3523	-0.1097	#####	0.0960	-0.2650	0.4621	-0.3260	-0.0966	-0.1444	0.2176	0.0193	0.0829	-0.0829	-0.2114	0.4166		-0.1815	-0.6838
Thigmotaxis %	0.4310	-0.0843	-0.4188	-0.2443	-0.2272	#####	0.1963	-0.0162	0.1244	-0.4231	-0.3120	-0.4084	0.3947	0.0159	-0.1560	0.3573	0.8487	0.6472	-0.1815		0.0983
Floating %	0.1287	-0.0599	-0.0170	-0.3839	-0.0200	0.0268	-0.2810	0.0952	-0.2338	0.2108	-0.0551	0.0226	-0.4336	0.2382	-0.0158	-0.0443	0.0952	-0.3781	-0.6838	0.0983	

P-Value	Weight	SVP Density	ICP Density	DCP Density	Total Retina	IRL	RPE	RNFL	IPL	INL	ORL	PRC	OPL	Number of Target Zone Crossings	% of time in sector with platform	Latency on Day 3	Latency on Day 4	Distance swam (m)	Velocity (m/s)	Thigmotaxis %	Floating %
Weight		0.1963	0.0601	0.8935	0.7273	0.7154	0.9899	0.7061	0.9620	0.8193	0.7637	0.8802	0.3295	0.5775	0.7806	0.3338	0.1179	0.3859	0.5205	0.1618	0.6903
SVP Density	0.1963		0.5530	0.8879	0.7785	0.3697	0.3299	0.6729	0.7492	0.8802	0.8482	0.7770	0.6593	0.5976	0.9853	0.5279	0.5591	0.6045	0.0469	0.7945	0.8534
ICP Density	0.0601	0.5530		0.1588	0.8588	0.8127	0.8926	0.5912	0.5940	0.2084	0.6398	0.8317	0.0866	0.1846	0.0588	0.3511	0.2427	0.3977	0.6056	0.1754	0.9582
DCP Density	0.8935	0.8879	0.1588		0.1128	0.0668	0.4792	0.3659	0.7839	0.0621	0.2166	0.1911	0.9182	0.4298	0.3452	0.3866	0.7286	0.7815	0.2614	0.4441	0.2179
Total Retina	0.7273	0.7785	0.8588	0.1128		0.0000	0.0734	0.0001	0.0081	0.0067	0.0000	0.0000	0.1186	0.2168	0.2114	0.9165	0.9899	0.9141	0.7344	0.4777	0.9508
IRL	0.7154	0.3697	0.8127	0.0668	0.0000		0.0426	0.0002	0.0379	0.0019	0.0024	0.0034	0.1927	0.3037	0.1564	0.7505	0.8518	0.9346	0.7162	0.7585	0.9342
RPE	0.9899	0.3299	0.8926	0.4792	0.0734	0.0426		0.0041	0.0556	0.8376	0.1556	0.3137	0.0010	0.0674	0.0441	0.9349	0.3091	0.2420	0.7665	0.5408	0.3763
RNFL	0.7061	0.6729	0.5912	0.3659	0.0001	0.0002	0.0041		0.0001	0.0482	0.0007	0.0023	0.0328	0.1124	0.1221	0.8384	0.4231	0.7566	0.4052	0.9600	0.7685
IPL	0.9620	0.7492	0.5940	0.7839	0.0081	0.0379	0.0556	0.0001		0.0893	0.0064	0.0089	0.1903	0.0663	0.1986	0.7708	0.4579	0.9812	0.1304	0.7001	0.4645
INL	0.8193	0.8802	0.2084	0.0621	0.0067	0.0019	0.8376	0.0482	0.0893		0.0348	0.0131	0.4952	0.3626	0.3330	0.5015	0.5112	0.2032	0.3010	0.1706	0.5107
ORL	0.7637	0.8482	0.6398	0.2166	0.0000	0.0024	0.1556	0.0007	0.0064	0.0348		0.0000	0.1156	0.2106	0.3205	0.9466	0.9017	0.9036	0.7653	0.3235	0.8649
PRC	0.8802	0.7770	0.8317	0.1911	0.0000	0.0034	0.3137	0.0023	0.0089	0.0131	0.0000		0.3007	0.2614	0.3746	0.9808	0.6733	0.6175	0.6544	0.1875	0.9444
OPL	0.3295	0.6593	0.0866	0.9182	0.1186	0.1927	0.0010	0.0328	0.1903	0.4952	0.1156	0.3007		0.2480	0.3416	0.8002	0.1018	0.0277	0.4968	0.2042	0.1591
Number of Target Zone Crossings	0.5775	0.5976	0.1846	0.4298	0.2168	0.3037	0.0674	0.1124	0.0663	0.3626	0.2106	0.2614	0.2480		0.0003	0.1278	0.2560	0.2514	0.9525	0.9608	0.4559
% of time in sector with platform	0.7806	0.9853	0.0588	0.3452	0.2114	0.1564	0.0441	0.1221	0.1986	0.3330	0.3205	0.3746	0.3416	0.0003		0.2012	0.2086	0.3349	0.7979	0.6282	0.9612
Latency on Day 3	0.3338	0.5279	0.3511	0.3866	0.9165	0.7505	0.9349	0.8384	0.7708	0.5015	0.9466	0.9808	0.8002	0.1278	0.2012		0.1668	0.2793	0.7979	0.2542	0.8913
Latency on Day 4	0.1179	0.5591	0.2427	0.7286	0.9899	0.8518	0.3091	0.4231	0.4579	0.5112	0.9017	0.6733	0.1018	0.2560	0.2086	0.1668		0.0023	0.5096	0.0005	0.7685
Distance swam (m)	0.3859	0.6045	0.3977	0.7815	0.9141	0.9346	0.2420	0.7566	0.9812	0.2032	0.9036	0.6175	0.0277	0.2514	0.3349	0.2793	0.0023		0.1779	0.0229	0.2256
Velocity (m/s)	0.5205	0.0469	0.6056	0.2614	0.7344	0.7162	0.7665	0.4052	0.1304	0.3010	0.7653	0.6544	0.4968	0.9525	0.7979	0.7979	0.5096	0.1779		0.5724	0.0142
Thigmotaxis %	0.1618	0.7945	0.1754	0.4441	0.4777	0.7585	0.5408	0.9600	0.7001	0.1706	0.3235	0.1875	0.2042	0.9608	0.6282	0.2542	0.0005	0.0229	0.5724		0.7611
Floating %	0.6903	0.8534	0.9582	0.2179	0.9508	0.9342	0.3763	0.7685	0.4645	0.5107	0.8649	0.9444	0.1591	0.4559	0.9612	0.8913	0.7685	0.2256	0.0142	0.7611	

Table 9: Correlation Coefficient and corresponding p-value for or all tested parameters for all non-transgenic animals. For the Correlation coefficient red indicates values from 0-0.3, yellow =0.3-0.5 and green 0.5-1. For the p-value red indicates values above 0.15. yellow values from 0.15-0.1 and green values from below 0.05.

R-Squared	Weight	SVP Density	ICP Density	DCP Density	Total Retina	IRL	RPE	RNFL	IPL	INL	ORL	PRC	OPL	Number of Target Zone Crossings	% of time in sector with platform	Latency on Day 3	Latency on Day 4	Distance swam (m)	Velocity (m/s)	Thigmotaxis %	Floating %
Weight		0.1468	0.2852	0.0017	0.0115	0.0126	0.0000	0.0134	0.0002	0.0050	0.0086	0.0022	0.0864	0.0291	0.0074	0.0935	0.2265	0.0760	0.0425	0.1858	0.0166
SVP Density	0.1468		0.0329	0.0019	0.0075	0.0737	0.0863	0.0168	0.0097	0.0022	0.0035	0.0076	0.0183	0.0262	0.0000	0.0410	0.0352	0.0278	0.3393	0.0071	0.0036
ICP Density	0.2852	0.0329		0.1720	0.0030	0.0053	0.0017	0.0271	0.0267	0.1397	0.0206	0.0043	0.2435	0.1541	0.2876	0.0873	0.1336	0.0724	0.0276	0.1754	0.0003
DCP Density	0.0017	0.0019	0.1720		0.2126	0.2733	0.0465	0.0748	0.0071	0.2816	0.1351	0.1500	0.0010	0.0576	0.0812	0.0758	0.0126	0.0081	0.1241	0.0597	0.1474
Total Retina	0.0115	0.0075	0.0030	0.2126		0.8433	0.2626	0.7835	0.4859	0.5024	0.9144	0.8859	0.2066	0.1350	0.1380	0.0012	0.0000	0.0012	0.0120	0.0516	0.0004
IRL	0.0126	0.0737	0.0053	0.2733	0.8433		0.3232	0.7305	0.3360	0.5979	0.5812	0.5567	0.1490	0.0957	0.1738	0.0106	0.0037	0.0007	0.0138	0.0099	0.0007
RPE	0.0000	0.0863	0.0017	0.0465	0.2626	0.3232		0.5420	0.2940	0.0040	0.1744	0.0920	0.6443	0.2723	0.3196	0.0007	0.1030	0.1340	0.0092	0.0386	0.0790
RNFL	0.0134	0.0168	0.0271	0.0748	0.7835	0.7305	0.5420		0.7824	0.3097	0.6651	0.5869	0.3512	0.2130	0.2032	0.0044	0.0652	0.0100	0.0702	0.0003	0.0091
IPL	0.0002	0.0097	0.0267	0.0071	0.4859	0.3360	0.2940	0.7824		0.2400	0.5058	0.4777	0.1505	0.2742	0.1454	0.0089	0.0563	0.0001	0.2135	0.0155	0.0547
INL	0.0050	0.0022	0.1397	0.2816	0.5024	0.5979	0.0040	0.3097	0.2400		0.3451	0.4423	0.0433	0.0758	0.0853	0.0464	0.0444	0.1564	0.1063	0.1790	0.0444
ORL	0.0086	0.0035	0.0206	0.1351	0.9144	0.5812	0.1744	0.6651	0.5058	0.3451		0.9749	0.2097	0.1385	0.0896	0.0005	0.0016	0.0015	0.0093	0.0973	0.0030
PRC	0.0022	0.0076	0.0043	0.1500	0.8859	0.5567	0.0920	0.5869	0.4777	0.4423	0.9749		0.0968	0.1130	0.0722	0.0001	0.0185	0.0259	0.0208	0.1668	0.0005
OPL	0.0864	0.0183	0.2435	0.0010	0.2066	0.1490	0.6443	0.3512	0.1505	0.0433	0.2097	0.0968		0.1192	0.0824	0.0067	0.2451	0.3984	0.0474	0.1558	0.1880
Number of Target Zone Crossings	0.0291	0.0262	0.1541	0.0576	0.1350	0.0957	0.2723	0.2130	0.2742	0.0758	0.1385	0.1130	0.1192		0.7160	0.2161	0.1268	0.1291	0.0004	0.0003	0.0567
% of time in sector with platform	0.0074	0.0000	0.2876	0.0812	0.1380	0.1738	0.3196	0.2032	0.1454	0.0853	0.0896	0.0722	0.0824	0.7160		0.1577	0.1530	0.0931	0.0069	0.0243	0.0002
Latency on Day 3	0.0935	0.0410	0.0873	0.0758	0.0012	0.0106	0.0007	0.0044	0.0089	0.0464	0.0005	0.0001	0.0067	0.2161	0.1577		0.1819	0.1157	0.0069	0.1276	0.0020
Latency on Day 4	0.2265	0.0352	0.1336	0.0126	0.0000	0.0037	0.1030	0.0652	0.0563	0.0444	0.0016	0.0185	0.2451	0.1268	0.1530	0.1819		0.6221	0.0447	0.7202	0.0091
Distance swam (m)	0.0760	0.0278	0.0724	0.0081	0.0012	0.0007	0.1340	0.0100	0.0001	0.1564	0.0015	0.0259	0.3984	0.1291	0.0931	0.1157	0.6221		0.1736	0.4188	0.1430
Velocity (m/s)	0.0425	0.3393	0.0276	0.1241	0.0120	0.0138	0.0092	0.0702	0.2135	0.1063	0.0093	0.0208	0.0474	0.0004	0.0069	0.0069	0.0447	0.1736		0.0329	0.4676
Thigmotaxis %	0.1858	0.0071	0.1754	0.0597	0.0516	0.0099	0.0386	0.0003	0.0155	0.1790	0.0973	0.1668	0.1558	0.0003	0.0243	0.1276	0.7202	0.4188	0.0329		0.0097
Floating %	0.0166	0.0036	0.0003	0.1474	0.0004	0.0007	0.0790	0.0091	0.0547	0.0444	0.0030	0.0005	0.1880	0.0567	0.0002	0.0020	0.0091	0.1430	0.4676	0.0097	

Mean Squared Error	Weight	SVP Density	ICP Density	DCP Density	Total Retina	IRL	RPE	RNFL	IPL	INL	ORL	PRC	OPL	Number of Target Zone Crossings	% of time in sector with platform	Latency on Day 3	Latency on Day 4	Distance swam (m)	Velocity (m/s)	Thigmotaxis %	Floating %
Weight		0.00016	0.00041	0.000274	76.5299	15.617	0.59757	27.5526	10.2599	2.88649	28.7205	25.2942	0.6554	4.986682692	479.5229385	148.80482	98.401248	28030.8751	7.132828	115.0455861	75.2779
SVP Density	0.00016		0.00056	0.000274	76.8398	14.651	0.546	27.4586	10.1628	2.89459	28.8676	25.1562	0.70427	5.001757528	483.0586565	157.42194	122.72754	29491.6385	4.921511	140.2907414	76.2708
ICP Density	0.000412	0.00056		0.000228	77.1875	15.731	0.59655	27.1721	9.98856	2.4957	28.3712	25.2402	0.54275	4.344833467	344.1300649	149.81971	110.21547	28138.5552	7.243281	116.5125935	76.5229
DCP Density	0.000274	0.00027	0.00023		60.9594	11.494	0.56979	25.8384	10.189	2.08385	25.0549	21.5471	0.7167	4.840483864	443.831838	151.71823	125.60926	30090.7867	6.524524	132.8605262	65.2613
Total Retina	76.52993	76.8398	77.1875	60.95938		2.4783	0.44066	6.04653	5.27599	1.44341	2.47829	2.89334	0.56917	4.44273019	416.4012432	163.96332	127.20634	30298.0036	7.359605	134.0028339	76.5144
IRL	15.61694	14.6506	15.7314	11.49356	2.47829		0.40442	7.52585	6.81394	1.16649	12.1317	11.2362	0.61052	4.644610404	399.1150958	162.41745	126.7427	30313.6313	7.346396	139.8980756	76.4901
RPE	0.597574	0.546	0.59655	0.569793	0.44066	0.4044		12.7915	7.24461	2.88927	23.9155	23.0166	0.25519	3.737598327	328.7024015	164.03791	114.10466	26271.1053	7.380469	135.8473944	70.5007
RNFL	27.55263	27.4586	27.1721	25.83842	6.04653	7.5258	12.7915		2.23342	2.00236	9.70263	10.4711	0.46543	4.042320943	384.9198214	163.43699	118.9118	30030.3375	6.926043	141.2572568	75.8509
IPL	10.25993	10.1628	9.98856	10.189	5.27599	6.8139	7.24461	2.23342		2.20472	14.3148	13.2403	0.60947	3.727811545	412.8319826	162.69445	120.05226	30333.3043	5.858556	139.1080189	72.3603
INL	2.886489	2.89459	2.4957	2.083855	1.44341	1.1665	2.88927	2.00236	2.20472		18.9718	14.1362	0.68636	4.746789699	441.8813846	156.54088	121.56586	25590.0178	6.657296	116.0025838	73.1428
ORL	28.72048	28.8676	28.3712	25.05489	2.47829	12.132	23.9155	9.70263	14.3148	18.9718		0.63706	0.56698	4.424992004	439.7997696	164.07567	127.00467	30288.3834	7.379705	127.54	76.3124
PRC	25.29419	25.1562	25.2402	21.54708	2.89334	11.236	23.0166	10.4711	13.2403	14.1362	0.63706		0.64794	4.555529353	448.1789808	164.143	124.85342	29550.2089	7.293909	117.7318306	76.5059
OPL	0.655398	0.70427	0.54275	0.716697	0.56917	0.6105	0.25519	0.46543	0.60947	0.68636	0.56698	0.64794		4.523987687	443.2676434	163.05148	96.034962	18249.189	7.096365	119.2821626	62.156
Number of Target Zone Crossings	4.986683	5.00176	4.34483	4.840484	4.44273	4.6446	3.7376	4.04232	3.72781	4.74679	4.42499	4.55553	4.52399		137.2105843	128.67644	111.08245	26419.6133	7.446406	141.2586586	72.2013
% of time in sector with platform	479.5229	483.059	344.13	443.8318	416.401	399.12	328.702	384.92	412.832	441.881	439.8	448.179	443.268	137.2105843		138.26403	107.74328	27511.4879	7.398029	137.8551199	76.526
Latency on Day 3	148.8048	157.422	149.82	151.7182	163.963	162.42	164.038	163.437	162.694	156.541	164.076	164.143	163.051	128.6764417	138.2640283		104.074	26825.2639	7.39804	123.2594164	76.3949
Latency on Day 4	98.40125	122.728	110.215	125.6093	127.206	126.74	114.105	118.912	120.052	121.566	127.005	124.853	96.035	111.0824495	107.7432762	104.074		11465.0993	7.116407	39.53110378	75.851
Distance swam (m)	28030.88	29491.6	28138.6	30090.79	30298	30314	26271.1	30030.3	30333.3	25590	30288.4	29550.2	18249.2	26419.61325	27511.48794	26825.264	11465.099		6.156232	82.11457998	65.6024
Velocity (m/s)	7.132828	4.92151	7.24328	6.524524	7.35961	7.3464	7.38047	6.92604	5.85856	6.6573	7.3797	7.29391	7.09636	7.446405878	7.398028872	7.3980398	7.116407	6.1562316		136.6407555	40.7526
Thigmotaxis %	115.0456	140.291	116.513	132.8605	134.003	139.9	135.847	141.257	139.108	116.003	127.54	117.732	119.282	141.2586586	137.8551199	123.25942	39.531104	82.11458	136.6408		75.8049
Floating %	75.27794	76.2708	76.5229	65.26129	76.5144	76.49	70.5007	75.8509	72.3603	73.1428	76.3124	76.5059	62.156	72.2013215	76.52596058	76.394895	75.851	65.602432	40.75259	75.80488212	

Table 10: R-squared and corresponding MSE for or all tested parameters for all non-transgenic animals. For the R-squared values red indicates values from 0-0.3, yellow =0.4-0.6 and green 0.6-1.

Correlation Coefficient	Weight	SVP Density	ICP Density	DCP Density	Total Retina	IRL	RPE	RNFL	IPL	INL	ORL	PRC	OPL	Number of Target Zone Crossings	% of time in sector with platform	Latency on Day 3	Latency on Day 4	Distance swam (m)	Velocity (m/s)	Thigmotaxis %	Floating %
Weight		-0.0697	-0.4180	0.0742	0.1425	0.1284	0.0354	0.1234	0.0021	-0.0673	0.1506	0.0821	0.4116	-0.1308	-0.1546	-0.2628	0.7773	0.6827	0.3230	0.4677	-0.0677
SVP Density	-0.0697		-0.6629	-0.1896	0.4870	0.4458	0.4984	0.5684	-0.3825	-0.0220	0.5093	0.4241	0.6147	0.3256	0.3603	-0.4007	-0.1175	0.0338	0.2928	-0.0184	-0.1023
ICP Density	-0.4180	-0.6629		0.4941	#####	0.0603	-0.1207	-0.1070	-0.0040	0.4627	-0.0690	0.0366	-0.5822	-0.5783	-0.6258	0.5159	-0.4132	-0.4658	-0.3342	-0.5138	0.0929
DCP Density	0.0742	-0.1896	0.4941		0.6410	0.7163	0.4235	0.5428	-0.3972	0.7086	0.5654	0.5631	0.1935	-0.7328	-0.7500	0.5606	0.1413	0.1244	0.0604	-0.1175	-0.4549
Total Retina	0.1425	0.4870	-0.0113	0.6410		0.9844	0.5311	0.9322	-0.7706	0.7852	0.9898	0.9723	0.4100	-0.3521	-0.3753	-0.0338	-0.1054	-0.0820	-0.0572	-0.3684	-0.0435
IRL	0.1284	0.4458	0.0603	0.7163	0.9844		0.5426	0.8919	-0.6770	0.7905	0.9493	0.9293	0.4106	-0.4040	-0.4332	0.0863	-0.0799	-0.0421	0.0443	-0.3223	-0.1538
RPE	0.0354	0.4984	-0.1207	0.4235	0.5311	0.5426		0.7545	-0.6223	0.0401	0.5097	0.3829	0.8362	-0.6014	-0.5305	-0.0266	0.2802	0.4003	0.5181	0.0635	-0.5718
RNFL	0.1234	0.5684	-0.1070	0.5428	0.9322	0.8919	0.7545		-0.8995	0.5760	0.9436	0.8872	0.6016	-0.4456	-0.4286	-0.1749	-0.0081	0.0369	0.0373	-0.3140	-0.1231
IPL	0.0021	-0.3825	-0.0040	-0.3972	#####	-0.6770	-0.6223	-0.8995		-0.5319	-0.8287	-0.8072	-0.3798	0.4342	0.4034	0.3203	0.1518	0.1609	0.2924	0.4973	-0.1349
INL	-0.0673	-0.0220	0.4627	0.7086	0.7852	0.7905	0.0401	0.5760	-0.5319		0.7629	0.8479	-0.2062	-0.3255	-0.3944	0.2035	-0.4062	-0.4652	-0.4753	-0.6241	0.2201
ORL	0.1506	0.5093	-0.0690	0.5654	0.9898	0.9493	0.5097	0.9436	-0.8287	0.7629		0.9850	0.4002	-0.3021	-0.3198	-0.1301	-0.1236	-0.1124	-0.1381	-0.3972	0.0468
PRC	0.0821	0.4241	0.0366	0.5631	0.9723	0.9293	0.3829	0.8872	-0.8072	0.8479	0.9850		0.2359	-0.2696	-0.3016	-0.1072	-0.2479	-0.2564	-0.2804	-0.5094	0.1668
OPL	0.4116	0.6147	-0.5822	0.1935	0.4100	0.4106	0.8362	0.6016	-0.3798	-0.2062	0.4002	0.2359		-0.2691	-0.1990	-0.1635	0.6199	0.7277	0.7105	0.4677	-0.6218
Number of Target Zone Crossings	-0.1308	0.3256	-0.5783	-0.7328	#####	-0.4040	-0.6014	-0.4456	0.4342	-0.3255	-0.3021	-0.2696	-0.2691		0.9917	-0.3430	-0.2641	-0.2474	-0.2022	0.1073	0.3502
% of time in sector with platform	-0.1546	0.3603	-0.6258	-0.7500	#####	-0.4332	-0.5305	-0.4286	0.4034	-0.3944	-0.3198	-0.3016	-0.1990	0.9917		-0.3648	-0.2261	-0.1994	-0.1693	0.1481	0.2869
Latency on Day 3	-0.2628	-0.4007	0.5159	0.5606	#####	0.0863	-0.0266	-0.1749	0.3203	0.2035	-0.1301	-0.1072	-0.1635	-0.3430	-0.3648		0.0834	0.1175	0.2019	0.2850	-0.4434
Latency on Day 4	0.7773	-0.1175	-0.4132	0.1413	#####	-0.0799	0.2802	-0.0081	0.1518	-0.4062	-0.1236	-0.2479	0.6199	-0.2641	-0.2261	0.0834		0.9754	0.6398	0.8380	-0.5558
Distance swam (m)	0.6827	0.0338	-0.4658	0.1244	#####	-0.0421	0.4003	0.0369	0.1609	-0.4652	-0.1124	-0.2564	0.7277	-0.2474	-0.1994	0.1175	0.9754		0.7776	0.8755	-0.6660
Velocity (m/s)	0.3230	0.2928	-0.3342	0.0604	#####	0.0443	0.5181	0.0373	0.2924	-0.4753	-0.1381	-0.2804	0.7105	-0.2022	-0.1693	0.2019	0.6398	0.7776		0.6960	-0.7515
Thigmotaxis %	0.4677	-0.0184	-0.5138	-0.1175	#####	-0.3223	0.0635	-0.3140	0.4973	-0.6241	-0.3972	-0.5094	0.4677	0.1073	0.1481	0.2850	0.8380	0.8755	0.6960		-0.5757
Floating %	-0.0677	-0.1023	0.0929	-0.4549	#####	-0.1538	-0.5718	-0.1231	-0.1349	0.2201	0.0468	0.1668	-0.6218	0.3502	0.2869	-0.4434	-0.5558	-0.6660	-0.7515	-0.5757	

P-Value	Weight	SVP Density	ICP Density	DCP Density	Total Retina	IRL	RPE	RNFL	IPL	INL	ORL	PRC	OPL	Number of Target Zone Crossings	% of time in sector with platform	Latency on Day 3	Latency on Day 4	Distance swam (m)	Velocity (m/s)	Thigmotaxis %	Floating %
Weight		0.8698	0.3027	0.8613	0.7365	0.7618	0.9336	0.7710	0.9961	0.8742	0.7219	0.8468	0.3110	0.7575	0.7148	0.5294	0.0232	0.0621	0.4352	0.2425	0.8734
SVP Density	0.8698		0.0732	0.6529	0.2210	0.2683	0.2087	0.1415	0.3497	0.9588	0.1974	0.2950	0.1048	0.4313	0.3806	0.3252	0.7817	0.9366	0.4816	0.9654	0.8094
ICP Density	0.3027	0.0732		0.2133	0.9788	0.8871	0.7758	0.8009	0.9925	0.2483	0.8710	0.9315	0.1300	0.1332	0.0970	0.1906	0.3089	0.2447	0.4184	0.1927	0.8269
DCP Density	0.8613	0.6529	0.2133		0.0868	0.0456	0.2958	0.1645	0.3298	0.0491	0.1441	0.1461	0.6461	0.0387	0.0321	0.1483	0.7385	0.7691	0.8870	0.7818	0.2574
Total Retina	0.7365	0.2210	0.9788	0.0868		0.0000	0.1756	0.0007	0.0252	0.0210	0.0000	0.0001	0.3131	0.3923	0.3596	0.9368	0.8038	0.8469	0.8929	0.3693	0.9186
IRL	0.7618	0.2683	0.8871	0.0456	0.0000		0.1646	0.0029	0.0651	0.0195	0.0003	0.0008	0.3123	0.3209	0.2836	0.8390	0.8508	0.9211	0.9170	0.4362	0.7162
RPE	0.9336	0.2087	0.7758	0.2958	0.1756	0.1646		0.0305	0.0994	0.9249	0.1969	0.3492	0.0097	0.1147	0.1762	0.9501	0.5014	0.3257	0.1884	0.8812	0.1386
RNFL	0.7710	0.1415	0.8009	0.1645	0.0007	0.0029	0.0305		0.0023	0.1351	0.0004	0.0033	0.1146	0.2685	0.2893	0.6787	0.9849	0.9308	0.9301	0.4489	0.7715
IPL	0.9961	0.3497	0.9925	0.3298	0.0252	0.0651	0.0994	0.0023		0.1748	0.0110	0.0154	0.3534	0.2824	0.3216	0.4393	0.7197	0.7034	0.4822	0.2098	0.7502
INL	0.8742	0.9588	0.2483	0.0491	0.0210	0.0195	0.9249	0.1351	0.1748		0.0277	0.0078	0.6241	0.4314	0.3336	0.6288	0.3180	0.2455	0.2340	0.0981	0.6004
ORL	0.7219	0.1974	0.8710	0.1441	0.0000	0.0003	0.1969	0.0004	0.0110	0.0277		0.0000	0.3259	0.4671	0.4400	0.7587	0.7706	0.7910	0.7443	0.3298	0.9124
PRC	0.8468	0.2950	0.9315	0.1461	0.0001	0.0008	0.3492	0.0033	0.0154	0.0078	0.0000		0.5739	0.5185	0.4678	0.8006	0.5538	0.5400	0.5012	0.1972	0.6930
OPL	0.3110	0.1048	0.1300	0.6461	0.3131	0.3123	0.0097	0.1146	0.3534	0.6241	0.3259	0.5739		0.5193	0.6365	0.6988	0.1011	0.0407	0.0482	0.2425	0.0998
Number of Target Zone Crossings	0.7575	0.4313	0.1332	0.0387	0.3923	0.3209	0.1147	0.2685	0.2824	0.4314	0.4671	0.5185	0.5193		0.0000	0.4055	0.5274	0.5547	0.6311	0.8003	0.3951
% of time in sector with platform	0.7148	0.3806	0.0970	0.0321	0.3596	0.2836	0.1762	0.2893	0.3216	0.3336	0.4400	0.4678	0.6365	0.0000		0.3743	0.5903	0.6358	0.6886	0.7263	0.4908
Latency on Day 3	0.5294	0.3252	0.1906	0.1483	0.9368	0.8390	0.9501	0.6787	0.4393	0.6288	0.7587	0.8006	0.6988	0.4055	0.3743		0.8444	0.7817	0.6316	0.4938	0.2711
Latency on Day 4	0.0232	0.7817	0.3089	0.7385	0.8038	0.8508	0.5014	0.9849	0.7197	0.3180	0.7706	0.5538	0.1011	0.5274	0.5903	0.8444		0.0000	0.0876	0.0094	0.1526
Distance swam (m)	0.0621	0.9366	0.2447	0.7691	0.8469	0.9211	0.3257	0.9308	0.7034	0.2455	0.7910	0.5400	0.0407	0.5547	0.6358	0.7817	0.0000		0.0231	0.0044	0.0714
Velocity (m/s)	0.4352	0.4816	0.4184	0.8870	0.8929	0.9170	0.1884	0.9301	0.4822	0.2340	0.7443	0.5012	0.0482	0.6311	0.6886	0.6316	0.0876	0.0231		0.0552	0.0316
Thigmotaxis %	0.2425	0.9654	0.1927	0.7818	0.3693	0.4362	0.8812	0.4489	0.2098	0.0981	0.3298	0.1972	0.2425	0.8003	0.7263	0.4938	0.0094	0.0044	0.0552		0.1353
Floating %	0.8734	0.8094	0.8269	0.2574	0.9186	0.7162	0.1386	0.7715	0.7502	0.6004	0.9124	0.6930	0.0998	0.3951	0.4908	0.2711	0.1526	0.0714	0.0316	0.1353	

Table 11: Correlation Coefficient and corresponding p-value for or all tested parameters for female non-transgenic animals. For the Correlation coefficient red indicates values from 0-0.3, yellow =0.3-0.5 and green 0.5-1. For the p-value red indicates values above 0.15, yellow values from 0.15-0.1 and green values from below 0.05.

R-Squared	Weight	SVP Density	ICP Density	DCP Density	Total Retina	IRL	RPE	RNFL	IPL	INL	ORL	PRC	OPL	Number of Target Zone Crossings	% of time in sector with platform	Latency on Day 3	Latency on Day 4	Distance swam (m)	Velocity (m/s)	Thigmotaxis %	Floating %
Weight		0.0049	0.1748	0.0055	0.0203	0.0165	0.0013	0.0152	0.0000	0.0045	0.0227	0.0067	0.1694	0.0171	0.0239	0.0691	0.6042	0.4661	0.1043	0.2187	0.0046
SVP Density	0.0049		0.4395	0.0360	0.2372	0.1987	0.2484	0.3231	0.1463	0.0005	0.2594	0.1799	0.3779	0.1060	0.1298	0.1606	0.0138	0.0011	0.0857	0.0003	0.0105
ICP Density	0.1748	0.4395		0.2441	0.0001	0.0036	0.0146	0.0114	0.0000	0.2141	0.0048	0.0013	0.3390	0.3344	0.3916	0.2661	0.1707	0.2170	0.1117	0.2640	0.0086
DCP Density	0.0055	0.0360	0.2441		0.4109	0.5131	0.1793	0.2947	0.1578	0.5021	0.3197	0.3171	0.0375	0.5369	0.5624	0.3143	0.0200	0.0155	0.0037	0.0138	0.2070
Total Retina	0.0203	0.2372	0.0001	0.4109		0.9690	0.2821	0.8690	0.5938	0.6165	0.9797	0.9454	0.1681	0.1240	0.1408	0.0011	0.0111	0.0067	0.0033	0.1357	0.0019
IRL	0.0165	0.1987	0.0036	0.5131	0.9690		0.2944	0.7954	0.4584	0.6249	0.9012	0.8636	0.1686	0.1632	0.1877	0.0074	0.0064	0.0018	0.0020	0.1039	0.0236
RPE	0.0013	0.2484	0.0146	0.1793	0.2821	0.2944		0.5693	0.3872	0.0016	0.2598	0.1466	0.6992	0.3617	0.2814	0.0007	0.0785	0.1603	0.2684	0.0040	0.3270
RNFL	0.0152	0.3231	0.0114	0.2947	0.8690	0.7954	0.5693		0.8091	0.3318	0.8904	0.7871	0.3619	0.1986	0.1837	0.0306	0.0001	0.0014	0.0014	0.0986	0.0151
IPL	0.0000	0.1463	0.0000	0.1578	0.5938	0.4584	0.3872	0.8091		0.2829	0.6868	0.6516	0.1442	0.1885	0.1628	0.1026	0.0231	0.0259	0.0855	0.2474	0.0182
INL	0.0045	0.0005	0.2141	0.5021	0.6165	0.6249	0.0016	0.3318	0.2829		0.5820	0.7189	0.0425	0.1060	0.1556	0.0414	0.1650	0.2164	0.2259	0.3895	0.0485
ORL	0.0227	0.2594	0.0048	0.3197	0.9797	0.9012	0.2598	0.8904	0.6868	0.5820		0.9702	0.1602	0.0913	0.1023	0.0169	0.0153	0.0126	0.0191	0.1578	0.0022
PRC	0.0067	0.1799	0.0013	0.3171	0.9454	0.8636	0.1466	0.7871	0.6516	0.7189	0.9702		0.0556	0.0727	0.0910	0.0115	0.0615	0.0657	0.0786	0.2595	0.0278
OPL	0.1694	0.3779	0.3390	0.0375	0.1681	0.1686	0.6992	0.3619	0.1442	0.0425	0.1602	0.0556		0.0724	0.0396	0.0267	0.3843	0.5295	0.5049	0.2188	0.3866
Number of Target Zone Crossings	0.0171	0.1060	0.3344	0.5369	0.1240	0.1632	0.3617	0.1986	0.1885	0.1060	0.0913	0.0727	0.0724		0.9834	0.1177	0.0697	0.0612	0.0409	0.0115	0.1226
% of time in sector with platform	0.0239	0.1298	0.3916	0.5624	0.1408	0.1877	0.2814	0.1837	0.1628	0.1556	0.1023	0.0910	0.0396	0.9834		0.1331	0.0511	0.0398	0.0287	0.0219	0.0823
Latency on Day 3	0.0691	0.1606	0.2661	0.3143	0.0011	0.0074	0.0007	0.0306	0.1026	0.0414	0.0169	0.0115	0.0267	0.1177	0.1331		0.0069	0.0138	0.0408	0.0812	0.1966
Latency on Day 4	0.6042	0.0138	0.1707	0.0200	0.0111	0.0064	0.0785	0.0001	0.0231	0.1650	0.0153	0.0615	0.3843	0.0697	0.0511	0.0069		0.9515	0.4093	0.7022	0.3089
Distance swam (m)	0.4661	0.0011	0.2170	0.0155	0.0067	0.0018	0.1603	0.0014	0.0259	0.2164	0.0126	0.0657	0.5295	0.0612	0.0398	0.0138	0.9515		0.6047	0.7665	0.4436
Velocity (m/s)	0.1043	0.0857	0.1117	0.0037	0.0033	0.0020	0.2684	0.0014	0.0855	0.2259	0.0191	0.0786	0.5049	0.0409	0.0287	0.0408	0.4093	0.6047		0.4844	0.5647
Thigmotaxis %	0.2187	0.0003	0.2640	0.0138	0.1357	0.1039	0.0040	0.0986	0.2474	0.3895	0.1578	0.2595	0.2188	0.0115	0.0219	0.0812	0.7022	0.7665	0.4844		0.3314
Floating %	0.0046	0.0105	0.0086	0.2070	0.0019	0.0236	0.3270	0.0151	0.0182	0.0485	0.0022	0.0278	0.3866	0.1226	0.0823	0.1966	0.3089	0.4436	0.5647	0.3314	

Mean Squared Error	Weight	SVP Density	ICP Density	DCP Density	Total Retina	IRL	RPE	RNFL	IPL	INL	ORL	PRC	OPL	Number of Target Zone Crossings	% of time in sector with platform	Latency on Day 3	Latency on Day 4	Distance swam (m)	Velocity (m/s)	Thigmotaxis %	Floating %
Weight		6.8E-05	0.00054	0.00033	114.86	23.6588	0.862778	37.7809	11.3469	4.39535	35.9048	32.4513	0.85832	4.530467446	430.5474464	128.59837	38.259146	19101.294	2.49839	96.23562141	61.83143
SVP Density	6.85E-05		0.00037	0.00032	89.431	19.2754	0.64927	25.9686	9.68704	4.41323	27.2098	26.794	0.64283	4.120843712	383.8181899	115.96273	95.333736	35736.384	2.55027	123.1391888	61.46589
ICP Density	0.000542	0.00037		0.00025	117.22	23.9679	0.851272	37.9259	11.3468	3.47007	36.5629	32.6278	0.68307	3.068006653	268.3711291	101.3796	80.162828	28013.128	2.4777	90.65708748	61.58069
DCP Density	0.000329	0.00032	0.00025		69.066	11.713	0.708946	27.0601	9.55656	2.19835	24.9916	22.3113	0.99463	2.134470562	192.9999314	94.72908	94.738303	35223.393	2.77915	121.481706	49.26072
Total Retina	114.8568	89.4308	117.221	69.0662		0.74461	0.620175	5.02456	4.60875	1.69346	0.74461	1.78401	0.85964	4.037901445	378.9651257	137.98483	95.595171	35536.73	2.7802	106.46692	61.99917
IRL	23.65875	19.2754	23.9679	11.713	0.7446		0.6095	7.84878	6.14572	1.65607	3.62893	4.4579	0.85916	3.857033267	358.2993982	137.11364	96.051403	35713.807	2.78385	110.3853575	60.64799
RPE	0.862778	0.64927	0.85127	0.70895	0.6202	0.6095		16.5255	6.95318	4.40826	27.1937	27.8827	0.3108	2.942012419	316.9430122	138.04439	89.077646	30043.586	2.04072	122.6840153	41.80575
RNFL	37.78088	25.9686	37.9259	27.0601	5.0246	7.84878	16.52546		2.16601	2.95049	4.02623	6.95467	0.65936	3.69414333	360.0412102	133.91594	96.662672	35728.482	2.78545	111.0396532	61.17548
IPL	11.34693	9.68704	11.3468	9.55656	4.6087	6.14572	6.953178	2.16601		3.16619	11.5057	11.3829	0.88432	3.74039742	369.2950716	123.97091	94.440352	34850.829	2.55087	92.71210358	60.98671
INL	4.395346	4.41323	3.47007	2.19835	1.6935	1.65607	4.408257	2.95049	3.16619		15.3562	9.18525	0.98939	4.120863508	372.4667858	132.41869	80.717873	28035.837	2.15932	75.19652745	59.10667
ORL	35.90483	27.2098	36.5629	24.9916	0.7446	3.62893	27.19375	4.02623	11.5057	15.3562		0.97511	0.86784	4.188746574	395.9665208	135.80297	95.192208	35325.326	2.73613	103.7445024	61.98061
PRC	32.45129	26.794	32.6278	22.3113	1.784	4.4579	27.8827	6.95467	11.3829	9.18525	0.97511		0.97586	4.274332485	400.9559016	136.55584	90.726905	33425.959	2.57004	91.21784972	60.38803
OPL	0.858319	0.64283	0.68307	0.99463	0.8596	0.85916	0.310799	0.65936	0.88432	0.98939	0.86784	0.97586		4.275618656	423.6114164	134.44798	59.518746	16832.84	1.3811	96.23367655	38.10244
Number of Target Zone Crossings	4.530467	4.12084	3.06801	2.13447	4.0379	3.85703	2.942012	3.69414	3.7404	4.12086	4.18875	4.27433	4.27562		7.324923099	121.88852	89.928769	33587.657	2.67528	121.7619291	54.49908
% of time in sector with platform	430.5474	383.818	268.371	193	378.97	358.299	316.943	360.041	369.295	372.467	395.967	400.956	423.611	7.324923099		119.76032	91.727196	34354.089	2.70941	120.4783246	57.00264
Latency on Day 3	128.5984	115.963	101.38	94.7291	137.98	137.114	138.0444	133.916	123.971	132.419	135.803	136.556	134.448	121.888523	119.7603249		95.997246	35283.369	2.67561	113.1748526	49.90308
Latency on Day 4	38.25915	95.3337	80.1628	94.7383	95.595	96.0514	89.07765	96.6627	94.4404	80.7179	95.1922	90.7269	59.5187	89.92876873	91.72719565	95.997246		1736.6452	1.64765	36.6882226	42.92695
Distance swam (m)	19101.29	35736.4	28013.1	35223.4	35537	35713.8	30043.59	35728.5	34850.8	28035.8	35325.3	33426	16832.8	33587.65701	34354.08881	35283.369	1736.6452		1.10268	28.76638276	34.56133
Velocity (m/s)	2.498387	2.55027	2.4777	2.77915	2.7802	2.78385	2.040716	2.78545	2.55087	2.15932	2.73613	2.57004	1.3811	2.675278376	2.709410625	2.6756122	1.6476509	1.1026779		63.5169528	27.04015
Thigmotaxis %	96.23562	123.139	90.6571	121.482	106.47	110.385	122.684	111.04	92.7121	75.1965	103.745	91.2178	96.2337	121.7619291	120.4783246	113.17485	36.688223	28.766383	63.517		41.52832
Floating %	61.83143	61.4659	61.5807	49.2607	61.999	60.648	41.80575	61.1755	60.9867	59.1067	61.9806	60.388	38.1024	54.49908376	57.00263726	49.903081	42.926953	34.56133	27.0401	41.52832057	

Table 12: R-squared and corresponding MSE for or all tested parameters all for female non-transgenic animals. For the R-squared values red indicates values from 0-0.3, yellow =0.4-0.6 and green 0.6-1.

Correlation Coefficient	Weight	SVP Density	ICP Density	DCP Density	Total Retina	IRL	RPE	RNFL	IPL	INL	ORL	PRC	OPL	Number of Target Zone Crossings	% of time in sector with platform	Latency on Day 3	Latency on Day 4	Distance swam (m)	Velocity (m/s)	Thigmotaxis %	Floating %
Weight		0.0375	0.0803	-0.0469	-0.0082	0.5634	0.4989	0.0221	0.3618	-0.2907	-0.2262	-0.2608	0.0826	0.5533	-0.2867	-0.4670	0.0655	-0.6240	-0.4541	0.5547	0.5589
SVP Density	0.0375		0.8573	0.4484	-0.9840	0.1649	-0.2584	-0.4144	0.4652	0.4884	-0.9652	-0.9434	-0.9253	0.6570	0.6595	-0.2450	-0.3491	-0.1391	0.5610	-0.1718	0.0950
ICP Density	0.0803	0.8573		0.6203	-0.9320	-0.1980	-0.4974	-0.8020	0.8186	0.1006	-0.7768	-0.7467	-0.8393	0.7940	0.8823	-0.6844	-0.7983	-0.5478	0.7877	-0.5662	-0.3706
DCP Density	-0.0469	0.4484	0.6203		-0.5366	-0.5903	-0.8587	-0.7840	0.7165	-0.4641	-0.2624	-0.1927	-0.7326	0.7196	0.7850	-0.2945	-0.5762	-0.1203	0.8301	-0.5198	-0.2686
Total Retina	-0.0082	-0.9840	-0.9320	-0.5366		-0.0150	0.3805	0.5661	-0.5891	-0.3771	0.9217	0.8938	0.9369	-0.7070	-0.7748	0.3938	0.5395	0.2613	-0.7024	0.3626	0.1092
IRL	0.5634	0.1649	-0.1980	-0.5903	-0.0150		0.8835	0.6741	-0.3767	0.5494	-0.4018	-0.4597	0.1197	-0.0220	-0.6135	0.2590	0.7437	0.0454	-0.9069	0.9749	0.9207
RPE	0.4989	-0.2584	-0.4974	-0.8587	0.3805	0.8835		0.7812	-0.5292	0.3862	0.0057	-0.0676	0.5572	-0.3410	-0.8352	0.1908	0.6730	-0.0360	-0.9741	0.8360	0.6626
RNFL	0.0221	-0.4144	-0.8020	-0.7840	0.5661	0.6741	0.7812		-0.9212	0.4426	0.2568	0.2092	0.5618	-0.6912	-0.9062	0.7454	0.9481	0.5799	-0.8971	0.8028	0.6655
IPL	0.3618	0.4652	0.8186	0.7165	-0.5891	-0.3767	-0.5292	-0.9212		-0.4654	-0.3933	-0.3624	-0.5437	0.8797	0.7561	-0.8608	-0.8485	-0.7652	0.6672	-0.5240	-0.3848
INL	-0.2907	0.4884	0.1006	-0.4641	-0.3771	0.5494	0.3862	0.4426	-0.4654		-0.5585	-0.5860	-0.2340	-0.2807	-0.0884	0.9799	0.9227	0.9087	-0.5764	0.6123	0.5804
ORL	-0.2262	-0.9652	-0.7768	-0.2624	0.9217	-0.4018	0.0057	0.2568	-0.3933	-0.5585		0.9970	0.8116	-0.6390	-0.4716	0.2815	0.1874	0.2513	-0.2753	-0.1163	-0.3547
PRC	-0.2608	-0.9434	-0.7467	-0.1927	0.8938	-0.4597	-0.0676	0.2092	-0.3624	-0.5860	0.9970		0.7639	-0.6117	-0.4164	0.2989	0.1482	0.2908	-0.1922	-0.1887	-0.4128
OPL	0.0826	-0.9253	-0.8393	-0.7326	0.9369	0.1197	0.5572	0.5618	-0.5437	-0.2340	0.8116	0.7639		-0.7102	-0.7885	0.1353	0.3638	-0.0083	-0.6727	0.2965	0.0244
Number of Target Zone Crossings	0.5533	0.6570	0.7940	0.7196	-0.7070	-0.0220	-0.3410	-0.6912	0.8797	-0.2807	-0.6390	-0.6117	-0.7102		0.6066	-0.6195	-0.5064	-0.5727	0.4285	-0.1304	0.0705
% of time in sector with platform	-0.2867	0.6595	0.8823	0.7850	-0.7748	-0.6135	-0.8352	-0.9062	0.7561	-0.0884	-0.4716	-0.4164	-0.7885	0.6066		-0.4883	-0.8297	-0.2860	0.9743	-0.8128	-0.6290
Latency on Day 3	-0.4670	-0.2450	-0.6844	-0.2945	0.3938	0.2590	0.1908	0.7454	-0.8608	0.9799	0.2815	0.2989	0.1353	-0.6195	-0.4883		0.8328	0.9736	-0.4019	0.4640	0.4643
Latency on Day 4	0.0655	-0.3491	-0.7983	-0.5762	0.5395	0.7437	0.6730	0.9481	-0.8485	0.9227	0.1874	0.1482	0.3638	-0.5064	-0.8297	0.8328		0.6869	-0.8227	0.8664	0.8124
Distance swam (m)	-0.6240	-0.1391	-0.5478	-0.1203	0.2613	0.0454	-0.0360	0.5799	-0.7652	0.9087	0.2513	0.2908	-0.0083	-0.5727	-0.2860	0.9736	0.6869		-0.1832	0.2655	0.2992
Velocity (m/s)	-0.4541	0.5610	0.7877	0.8301	-0.7024	-0.9069	-0.9741	-0.8971	0.6672	-0.5764	-0.2753	-0.1922	-0.6727	0.4285	0.9743	-0.4019	-0.8227	-0.1832		-0.9059	-0.7554
Thigmotaxis %	0.5547	-0.1718	-0.5662	-0.5198	0.3626	0.9749	0.8360	0.8028	-0.5240	0.6123	-0.1163	-0.1887	0.2965	-0.1304	-0.8128	0.4640	0.8664	0.2655	-0.9059		0.9618
Floating %	0.5589	0.0950	-0.3706	-0.2686	0.1092	0.9207	0.6626	0.6655	-0.3848	0.5804	-0.3547	-0.4128	0.0244	0.0705	-0.6290	0.4643	0.8124	0.2992	-0.7554	0.9618	

P-Value	Weight	SVP Density	ICP Density	DCP Density	Total Retina	IRL	RPE	RNFL	IPL	INL	ORL	PRC	OPL	Number of Target Zone Crossings	% of time in sector with platform	Latency on Day 3	Latency on Day 4	Distance swam (m)	Velocity (m/s)	Thigmotaxis %	Floating %
Weight		0.9522	0.8979	0.9403	0.9895	0.3227	0.3922	0.9719	0.5496	0.6351	0.7145	0.6718	0.8949	0.3333	0.6400	0.5330	0.9345	0.3760	0.5459	0.4453	0.4411
SVP Density	0.9522		0.0633	0.4488	0.0024	0.7910	0.6746	0.4879	0.4298	0.4038	0.0078	0.0160	0.0242	0.2283	0.2259	0.7550	0.6509	0.8609	0.4390	0.8282	0.9050
ICP Density	0.8979	0.0633		0.2642	0.0211	0.7495	0.3939	0.1026	0.0902	0.8721	0.1223	0.1471	0.0754	0.1087	0.0476	0.3156	0.2017	0.4522	0.2123	0.4338	0.6294
DCP Density	0.9403	0.4488	0.2642		0.3512	0.2947	0.0624	0.1165	0.1733	0.4311	0.6698	0.7562	0.1591	0.1706	0.1157	0.7055	0.4238	0.8797	0.1699	0.4802	0.7314
Total Retina	0.9895	0.0024	0.0211	0.3512		0.9809	0.5274	0.3199	0.2959	0.5315	0.0260	0.0409	0.0189	0.1818	0.1238	0.6062	0.4605	0.7387	0.2976	0.6374	0.8908
IRL	0.3227	0.7910	0.7495	0.2947	0.9809		0.0469	0.2120	0.5319	0.3374	0.5026	0.4361	0.8480	0.9720	0.2711	0.7410	0.2563	0.9546	0.0931	0.0251	0.0793
RPE	0.3922	0.6746	0.3939	0.0624	0.5274	0.0469		0.1188	0.3591	0.5207	0.9928	0.9139	0.3292	0.5743	0.0783	0.8092	0.3270	0.9640	0.0259	0.1640	0.3374
RNFL	0.9719	0.4879	0.1026	0.1165	0.3199	0.2120	0.1188		0.0262	0.4555	0.6766	0.7356	0.3244	0.1962	0.0340	0.2546	0.0519	0.4201	0.1029	0.1972	0.3345
IPL	0.5496	0.4298	0.0902	0.1733	0.2959	0.5319	0.3591	0.0262		0.4296	0.5124	0.5489	0.3435	0.0491	0.1392	0.1392	0.1515	0.2348	0.3328	0.4760	0.6152
INL	0.6351	0.4038	0.8721	0.4311	0.5315	0.3374	0.5207	0.4555	0.4296		0.3278	0.2992	0.7049	0.6473	0.8875	0.0201	0.0773	0.0913	0.4236	0.3877	0.4196
ORL	0.7145	0.0078	0.1223	0.6698	0.0260	0.5026	0.9928	0.6766	0.5124	0.3278		0.0002	0.0954	0.2458	0.4226	0.7185	0.8126	0.7487	0.7247	0.8837	0.6453
PRC	0.6718	0.0160	0.1471	0.7562	0.0409	0.4361	0.9139	0.7356	0.5489	0.2992	0.0002		0.1327	0.2729	0.4856	0.7011	0.8518	0.7092	0.8078	0.8113	0.5872
OPL	0.8949	0.0242	0.0754	0.1591	0.0189	0.8480	0.3292	0.3244	0.3435	0.7049	0.0954	0.1327		0.1789	0.1130	0.8647	0.6362	0.9917	0.3273	0.7035	0.9756
Number of Target Zone Crossings	0.3333	0.2283	0.1087	0.1706	0.1818	0.9720	0.5743	0.1962	0.0491	0.6473	0.2458	0.2729	0.1789		0.2781	0.3805	0.4936	0.4273	0.5715	0.8696	0.9295
% of time in sector with platform	0.6400	0.2259	0.0476	0.1157	0.1238	0.2711	0.0783	0.0340	0.1392	0.8875	0.4226	0.4856	0.1130	0.2781		0.5117	0.1703	0.7140	0.0257	0.1872	0.3710
Latency on Day 3	0.5330	0.7550	0.3156	0.7055	0.6062	0.7410	0.8092	0.2546	0.1392	0.0201	0.7185	0.7011	0.8647	0.3805	0.5117		0.1672	0.0264	0.5981	0.5360	0.5357
Latency on Day 4	0.9345	0.6509	0.2017	0.4238	0.4605	0.2563	0.3270	0.0519	0.1515	0.0773	0.8126	0.8518	0.6362	0.4936	0.1703	0.1672		0.3131	0.1773	0.1336	0.1876
Distance swam (m)	0.3760	0.8609	0.4522	0.8797	0.7387	0.9546	0.9640	0.4201	0.2348	0.0913	0.7487	0.7092	0.9917	0.4273	0.7140	0.0264	0.3131		0.8168	0.7345	0.7008
Velocity (m/s)	0.5459	0.4390	0.2123	0.1699	0.2976	0.0931	0.0259	0.1029	0.3328	0.4236	0.7247	0.8078	0.3273	0.5715	0.0257	0.5981	0.1773	0.8168		0.0941	0.2446
Thigmotaxis %	0.4453	0.8282	0.4338	0.4802	0.6374	0.0251	0.1640	0.1972	0.4760	0.3877	0.8837	0.8113	0.7035	0.8696	0.1872	0.5360	0.1336	0.7345	0.0941		0.0382
Floating %	0.4411	0.9050	0.6294	0.7314	0.8908	0.0793	0.3374	0.3345	0.6152	0.4196	0.6453	0.5872	0.9756	0.9295	0.3710	0.5357	0.1876	0.7008	0.2446	0.0382	

Table 13: Correlation Coefficient and corresponding p-value for or all tested parameters for male non-transgenic animals. For the Correlation coefficient red indicates values from 0-0.3, yellow =0.3-0.5 and green 0.5-1. For the p-value red indicates values above 0.15, yellow values from 0.15-0.1 and green values from below 0.05.

R-Squared	Weight	SVP Density	ICP Density	DCP Density	Total Retina	IRL	RPE	RNFL	IPL	INL	ORL	PRC	OPL	Number of Target Zone Crossings	% of time in sector with platform	Latency on Day 3	Latency on Day 4	Distance swam (m)	Velocity (m/s)	Thigmotaxis %	Floating %
Weight		0.0014	0.0064	0.0022	0.0001	0.3174	0.2489	0.0005	0.1309	0.0845	0.0511	0.0680	0.0068	0.3062	0.0822	0.2181	0.0043	0.3894	0.2062	0.3076	0.3124
SVP Density	0.0014		0.7350	0.2011	0.9682	0.0272	0.0668	0.1717	0.2164	0.2386	0.9316	0.8901	0.8561	0.4317	0.4349	0.0600	0.1219	0.0193	0.3147	0.0295	0.0090
ICP Density	0.0064	0.7350		0.3848	0.8687	0.0392	0.2474	0.6432	0.6701	0.0101	0.6033	0.5576	0.7045	0.6304	0.7785	0.4685	0.6372	0.3001	0.6204	0.3205	0.1373
DCP Density	0.0022	0.2011	0.3848		0.2879	0.3484	0.7373	0.6147	0.5133	0.2153	0.0688	0.0371	0.5367	0.5178	0.6162	0.0867	0.3319	0.0145	0.6891	0.2702	0.0721
Total Retina	0.0001	0.9682	0.8687	0.2879		0.0002	0.1448	0.3204	0.3470	0.1422	0.8495	0.7990	0.8777	0.4999	0.6004	0.1551	0.2910	0.0683	0.4934	0.1315	0.0119
IRL	0.3174	0.0272	0.0392	0.3484	0.0002		0.7806	0.4545	0.1419	0.3019	0.1614	0.2113	0.0143	0.0005	0.3763	0.0671	0.5531	0.0021	0.8224	0.9503	0.8477
RPE	0.2489	0.0668	0.2474	0.7373	0.1448	0.7806		0.6102	0.2801	0.1492	0.0000	0.0046	0.3105	0.1163	0.6976	0.0364	0.4529	0.0013	0.9489	0.6989	0.4390
RNFL	0.0005	0.1717	0.6432	0.6147	0.3204	0.4545	0.6102		0.8486	0.1959	0.0660	0.0438	0.3156	0.4777	0.8212	0.5556	0.8989	0.3363	0.8049	0.6445	0.4429
IPL	0.1309	0.2164	0.6701	0.5133	0.3470	0.1419	0.2801	0.8486		0.2166	0.1547	0.1313	0.2956	0.7740	0.5717	0.7410	0.7199	0.5856	0.4451	0.2746	0.1480
INL	0.0845	0.2386	0.0101	0.2153	0.1422	0.3019	0.1492	0.1959	0.2166		0.3120	0.3433	0.0547	0.0788	0.0078	0.9602	0.8513	0.8258	0.3323	0.3749	0.3369
ORL	0.0511	0.9316	0.6033	0.0688	0.8495	0.1614	0.0000	0.0660	0.1547	0.3120		0.9940	0.6587	0.4083	0.2224	0.0792	0.0351	0.0632	0.0758	0.0135	0.1258
PRC	0.0680	0.8901	0.5576	0.0371	0.7990	0.2113	0.0046	0.0438	0.1313	0.3433	0.9940		0.5835	0.3742	0.1734	0.0893	0.0220	0.0845	0.0369	0.0356	0.1704
OPL	0.0068	0.8561	0.7045	0.5367	0.8777	0.0143	0.3105	0.3156	0.2956	0.0547	0.6587	0.5835		0.5043	0.6217	0.0183	0.1323	0.0001	0.4525	0.0879	0.0006
Number of Target Zone Crossings	0.3062	0.4317	0.6304	0.5178	0.4999	0.0005	0.1163	0.4777	0.7740	0.0788	0.4083	0.3742	0.5043		0.3680	0.3838	0.2565	0.3280	0.1836	0.0170	0.0050
% of time in sector with platform	0.0822	0.4349	0.7785	0.6162	0.6004	0.3763	0.6976	0.8212	0.5717	0.0078	0.2224	0.1734	0.6217	0.3680		0.2385	0.6884	0.0818	0.9493	0.6607	0.3957
Latency on Day 3	0.2181	0.0600	0.4685	0.0867	0.1551	0.0671	0.0364	0.5556	0.7410	0.9602	0.0792	0.0893	0.0183	0.3838	0.2385		0.6935	0.9480	0.1615	0.2153	0.2156
Latency on Day 4	0.0043	0.1219	0.6372	0.3319	0.2910	0.5531	0.4529	0.8989	0.7199	0.8513	0.0351	0.0220	0.1323	0.2565	0.6884	0.6935		0.4718	0.6768	0.7507	0.6599
Distance swam (m)	0.3894	0.0193	0.3001	0.0145	0.0683	0.0021	0.0013	0.3363	0.5856	0.8258	0.0632	0.0845	0.0001	0.3280	0.0818	0.9480	0.4718		0.0336	0.0705	0.0895
Velocity (m/s)	0.2062	0.3147	0.6204	0.6891	0.4934	0.8224	0.9489	0.8049	0.4451	0.3323	0.0758	0.0369	0.4525	0.1836	0.9493	0.1615	0.6768	0.0336		0.8206	0.5706
Thigmotaxis %	0.3076	0.0295	0.3205	0.2702	0.1315	0.9503	0.6989	0.6445	0.2746	0.3749	0.0135	0.0356	0.0879	0.0170	0.6607	0.2153	0.7507	0.0705	0.8206		0.9251
Floating %	0.3124	0.0090	0.1373	0.0721	0.0119	0.8477	0.4390	0.4429	0.1480	0.3369	0.1258	0.1704	0.0006	0.0050	0.3957	0.2156	0.6599	0.0895	0.5706	0.9251	

Mean Squared Error	Weight	SVP Density	ICP Density	DCP Density	Total Retina	IRL	RPE	RNFL	IPL	INL	ORL	PRC	OPL	Number of Target Zone Crossings	% of time in sector with platform	Latency on Day 3	Latency on Day 4	Distance swam (m)	Velocity (m/s)	Thigmotaxis %	Floating %
Weight		0.00014	5.09E-05	0.0001851	13.7135	1.68062	0.10007	11.0619	6.78803	0.4363	15.5142	12.489	0.20826	3.607894737	285.0386308	166.606	187.1345	7851.7885	9.61774	122.5694663	71.65259
SVP Density	0.000136		1.36E-05	0.0001482	0.43617	2.3951	0.12432	9.16705	6.12029	0.36288	1.1191	1.47274	0.03017	2.955200041	175.4849022	200.2779	165.0327	12609.977	8.303371	171.8052101	103.2611
ICP Density	5.09E-05	1.4E-05		0.0001141	1.80086	2.36552	0.10027	3.94932	2.57671	0.47175	6.48554	5.92849	0.06197	1.921974568	68.795818	113.2558	68.18276	8999.8975	4.599234	120.2854882	89.89221
DCP Density	0.000185	0.00015	0.000114		9.76581	1.6042	0.035	4.26436	3.80103	0.37395	15.225	12.9028	0.09714	2.507679877	119.1871817	194.5872	125.5532	12672.459	3.766591	129.1923886	96.68517
Total Retina	13.71354	0.43617	1.800865	9.7658052		2.46153	0.11393	7.52111	5.10023	0.40881	2.46153	2.694	0.02564	2.600506435	124.1114943	180.0258	133.2438	11980.449	6.138515	153.7604597	102.9595
IRL	1.680616	2.3951	2.365516	1.6042044	2.46153		0.02922	6.03769	6.70204	0.33272	13.7114	10.569	0.20669	5.197490939	193.6867397	198.7718	83.99595	12832.235	2.151644	8.792553498	15.8656
RPE	0.100066	0.12432	0.100266	0.0349955	0.11393	0.02922		4.31401	5.62316	0.40548	16.35	13.3389	0.14458	4.595163707	93.90947675	205.3091	102.8289	12842.044	0.61887	53.30400895	58.4559
RNFL	11.06188	9.16705	3.949321	4.2643623	7.52111	6.03769	4.31401		1.18249	0.38322	15.272	12.8138	0.14352	2.715896664	55.54191597	94.68775	19.00193	8534.4454	2.364227	62.93140751	58.05304
IPL	6.788029	6.12029	2.576708	3.801028	5.10023	6.70204	5.62316	1.18249		0.37336	13.8212	11.6407	0.1477	1.175433716	133.0194146	55.1854	52.63283	5329.023	6.723252	128.4159372	88.77589
INL	0.436296	0.36288	0.471754	0.3739473	0.40881	0.33272	0.40548	0.38322	0.37336		11.2496	8.79943	0.19822	4.79025151	308.1344424	8.470873	27.93961	2240.4087	8.09033	110.6616624	69.09802
ORL	15.51421	1.1191	6.485539	15.224985	2.46153	13.7114	16.35	15.272	13.8212	11.2496		0.08042	0.07158	3.076624377	241.4989548	196.1867	181.3422	12046.608	11.19804	174.6384606	91.08837
PRC	12.48899	1.47274	5.928493	12.902799	2.694	10.569	13.3389	12.8138	11.6407	8.79943	0.08042		0.08733	3.254308248	256.7272547	194.035	183.8103	11771.587	11.66862	170.7251365	86.44578
OPL	0.208265	0.03017	0.061974	0.0971426	0.02564	0.20669	0.14458	0.14352	0.1477	0.19822	0.07158	0.08733		2.577425163	117.475555	209.1671	163.0667	12857.815	6.633848	161.4655513	104.1394
Number of Target Zone Crossings	3.607895	2.9552	1.921975	2.5076799	2.60051	5.19749	4.59516	2.7159	1.17543	4.79025	3.07662	3.25431	2.57743		196.2893941	131.2954	139.7386	8641.0985	9.891138	174.0216512	103.6843
% of time in sector with platform	285.0386	175.485	68.79582	119.18718	124.111	193.687	93.9095	55.5419	133.019	308.134	241.499	256.727	117.476	196.2893941		162.2582	58.5615	11807.12	0.613792	60.07134553	62.96898
Latency on Day 3	166.606	200.278	113.2558	194.58723	180.026	198.772	205.309	94.6877	55.1854	8.47087	196.187	194.035	209.167	131.2954487	162.258232		57.59861	668.99503	10.1587	138.9239041	81.73392
Latency on Day 4	187.1345	165.033	68.18276	125.55321	133.244	83.9959	102.829	19.0019	52.6328	27.9396	181.342	183.81	163.067	139.7386266	58.56149891	57.59861		6791.6285	3.915639	44.13628579	35.43657
Distance swam (m)	7851.789	12610	8999.898	12672.459	11980.4	12832.2	12842	8534.45	5329.02	2240.41	12046.6	11771.6	12857.8	8641.098471	11807.12008	668.995	6791.628		11.70951	164.5501029	94.87433
Velocity (m/s)	9.61774	8.30337	4.599234	3.7665908	6.13851	2.15164	0.61887	2.36423	6.72325	8.09033	11.198	11.6686	6.63385	9.891138456	0.613792428	10.1587	3.915639	11.709514		31.75477952	44.74547
Thigmotaxis %	122.5695	171.805	120.2855	129.19239	153.76	8.79255	53.304	62.9314	128.416	110.662	174.638	170.725	161.466	174.0216512	60.07134553	138.9239	44.13629	164.5501	31.75478		7.809298
Floating %	71.65259	103.261	89.89221	96.685174	102.96	15.8656	58.4559	58.053	88.7759	69.098	91.0884	86.4458	104.139	103.6843105	62.96897569	81.73392	35.43657	94.874331	44.74547	7.809298045	

Table 14: R-squared and corresponding MSE for or all tested parameters all for male non-transgenic animals. For the R-squared values red indicates values from 0-0.3, yellow =0.4-0.6 and green 0.6-1.
